# AI Quality Standards in Health Care: Rapid Umbrella Review

**DOI:** 10.2196/54705

**Published:** 2024-05-22

**Authors:** Craig E Kuziemsky, Dillon Chrimes, Simon Minshall, Michael Mannerow, Francis Lau

**Affiliations:** 1 MacEwan University Edmonton, AB Canada; 2 School of Health Information Science University of Victoria Victoria, BC Canada

**Keywords:** artificial intelligence, health care artificial intelligence, health care AI, rapid review, umbrella review, quality standard

## Abstract

**Background:**

In recent years, there has been an upwelling of artificial intelligence (AI) studies in the health care literature. During this period, there has been an increasing number of proposed standards to evaluate the quality of health care AI studies.

**Objective:**

This rapid umbrella review examines the use of AI quality standards in a sample of health care AI systematic review articles published over a 36-month period.

**Methods:**

We used a modified version of the Joanna Briggs Institute umbrella review method. Our rapid approach was informed by the practical guide by Tricco and colleagues for conducting rapid reviews. Our search was focused on the MEDLINE database supplemented with Google Scholar. The inclusion criteria were English-language systematic reviews regardless of review type, with mention of AI and health in the abstract, published during a 36-month period. For the synthesis, we summarized the AI quality standards used and issues noted in these reviews drawing on a set of published health care AI standards, harmonized the terms used, and offered guidance to improve the quality of future health care AI studies.

**Results:**

We selected 33 review articles published between 2020 and 2022 in our synthesis. The reviews covered a wide range of objectives, topics, settings, designs, and results. Over 60 AI approaches across different domains were identified with varying levels of detail spanning different AI life cycle stages, making comparisons difficult. Health care AI quality standards were applied in only 39% (13/33) of the reviews and in 14% (25/178) of the original studies from the reviews examined, mostly to appraise their methodological or reporting quality. Only a handful mentioned the transparency, explainability, trustworthiness, ethics, and privacy aspects. A total of 23 AI quality standard–related issues were identified in the reviews. There was a recognized need to standardize the planning, conduct, and reporting of health care AI studies and address their broader societal, ethical, and regulatory implications.

**Conclusions:**

Despite the growing number of AI standards to assess the quality of health care AI studies, they are seldom applied in practice. With increasing desire to adopt AI in different health topics, domains, and settings, practitioners and researchers must stay abreast of and adapt to the evolving landscape of health care AI quality standards and apply these standards to improve the quality of their AI studies.

## Introduction

### Growth of Health Care Artificial Intelligence

In recent years, there has been an upwelling of artificial intelligence (AI)–based studies in the health care literature. While there have been reported benefits, such as improved prediction accuracy and monitoring of diseases [1], health care organizations face potential patient safety, ethical, legal, social, and other risks from the adoption of AI approaches [2,3]. A search of the MEDLINE database for the terms “artificial intelligence” and “health” in the abstracts of articles published in 2022 alone returned >1000 results. Even by narrowing it down to systematic review articles, the same search returned dozens of results. These articles cover a wide range of AI approaches applied in different health care contexts, including such topics as the application of machine learning (ML) in skin cancer [4], use of natural language processing (NLP) to identify atrial fibrillation in electronic health records [5], image-based AI in inflammatory bowel disease [6], and predictive modeling of pressure injury in hospitalized patients [7]. The AI studies reported are also at different AI life cycle stages, from model development, validation, and deployment to evaluation [8]. Each of these AI life cycle stages can involve different contexts, questions, designs, measures, and outcomes [9]. With the number of health care AI studies rapidly on the rise, there is a need to evaluate the quality of these studies in different contexts. However, the means to examine the quality of health care AI studies have grown more complex, especially when considering their broader societal and ethical implications [10-13].

Coiera et al [14] described a “replication crisis” in health and biomedical informatics where issues regarding experimental design and reporting of results impede our ability to replicate existing research. Poor replication raises concerns about the quality of published studies as well as the ability to understand how context could impact replication across settings. The replication issue is prevalent in health care AI studies as many are single-setting approaches and we do not know the extent to which they can be translated to other settings or contexts. One solution to address the replication issue in AI studies has been the development of a growing number of AI quality standards. Most prominent are the reporting guidelines from the Enhancing the Quality and Transparency of Health Research (EQUATOR) network [15]. Examples include the CONSORT-AI (Consolidated Standards of Reporting Trials–Artificial Intelligence) extension for reporting AI clinical trials [16] and the SPIRIT-AI (Standard Protocol Items: Recommendations for Interventional Trials–Artificial Intelligence) extension for reporting AI clinical trial protocols [17]. Beyond the EQUATOR guidelines, there are also the Minimum Information for Medical AI Reporting standard [18] and the Minimum Information About Clinical Artificial Intelligence Modeling checklist [19] on the minimum information needed in published AI studies. These standards mainly focus on the methodological and reporting quality aspects of AI studies to ensure that the published information is rigorous, complete, and transparent.

### Need for Health Care AI Standards

However, there is a shortcoming of standard-driven guidance that spans the entire AI life cycle spectrum of design, validation, implementation, and governance. The World Health Organization has published six ethical principles to guide the use of AI [20] that cover (1) protecting human autonomy; (2) promoting human well-being and safety and the public interest; (3) ensuring transparency, explainability, and intelligibility; (4) fostering responsibility and accountability; (5) ensuring inclusiveness and equity; and (6) promoting AI that is responsive and sustainable. In a scoping review, Solanki et al [21] operationalized health care AI ethics through a framework of 6 guidelines that spans the entire AI life cycle of data management, model development, deployment, and monitoring. The National Health Service England has published a best practice guide on health care AI on *how to get it right* that encompasses a governance framework, addressing data access and protection issues, spreading the good innovation, and monitoring uses over time [22]. To further promote the quality of health care AI, van de Sande et al [23] have proposed a step-by-step approach with specific AI quality criteria that span the entire AI life cycle from development and implementation to governance.

Despite the aforementioned principles, frameworks, and guidance, there is still widespread variation in the quality of published AI studies in the health care literature. For example, 2 systematic reviews of 152 prediction and 28 diagnosis studies have shown poor methodological and reporting quality that have made it difficult to replicate, assess, and interpret the study findings [24,25]. The recent shifts beyond study quality to broader ethical, equity, and regulatory issues have also raised additional challenges for AI practitioners and researchers on the impact, transparency, trustworthiness, and accountability of the AI studies involved [13,26-28]. Increasingly, we are also seeing reports of various types of AI implementation issues [2]. There is a growing gap between the expected quality and performance of health care AI that needs to be addressed. We suggest that the overall issue is a lack of awareness and of the use of principles, frameworks, and guidance in health care AI studies.

This rapid umbrella review addressed the aforementioned issues by focusing on the principles and frameworks for health care AI design, implementation, and governance. We analyzed and synthesized the use of AI quality standards as reported in a sample of published health care AI systematic review articles. In this paper, AI quality standards are defined as guidelines, criteria, checklists, statements, guiding principles, or framework components used to evaluate the quality of health care AI studies in different domains and life cycle stages. In this context, quality covers the trustworthiness, methodological, reporting, and technical aspects of health care AI studies. Domains refer to the disciplines, branches, or areas in which AI can be found or applied, such as computer science, medicine, and robotics. The findings from this review can help address the growing need for AI practitioners and researchers to navigate the increasingly complex landscape of AI quality standards to plan, conduct, evaluate, and report health care AI studies.

## Methods

### Overview

**Figure 1:**
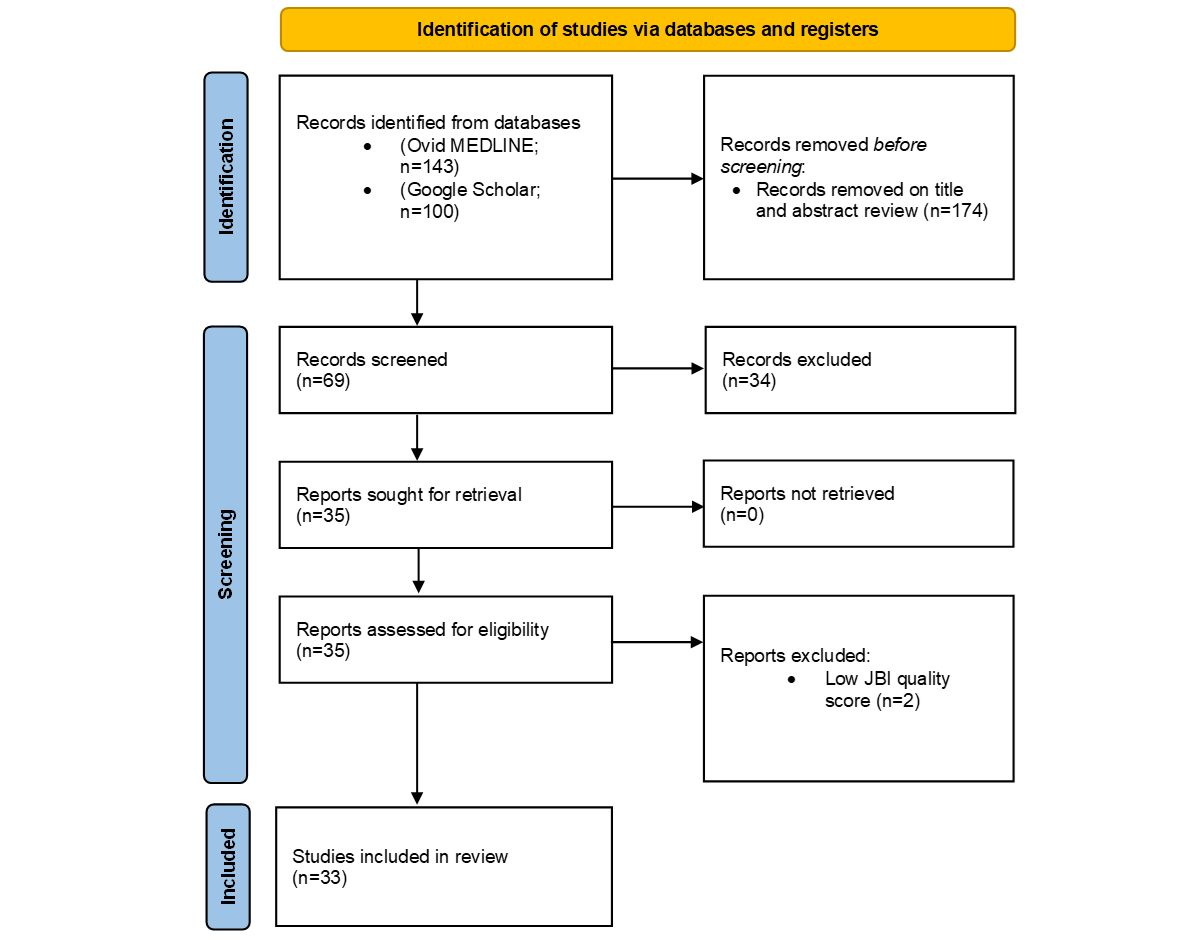
PRISMA (Preferred Reporting Items for Systematic Reviews and Meta-Analyses) flow diagram based on the work by Page et al [32]. JBI: Joanna Briggs Institute.

With the increasing volume of systematic review articles that appear in the health care literature each year, an umbrella review has become a popular and timely approach to synthesize knowledge from published systematic reviews on a given topic. For this paper, we drew on the umbrella review method in the typology of systematic reviews for synthesizing evidence in health care by MacEntee [29]. In this typology, umbrella reviews are used to synthesize multiple systematic reviews from different sources into a summarized form to address a specific topic. We used a modified version of the Joanna Briggs Institute (JBI) umbrella review method to tailor the process, including developing of an umbrella review protocol, applying a rapid approach, and eliminating duplicate original studies [30]. Our rapid approach was informed by the practical guide to conducting rapid reviews in the areas of database selection, topic refinement, searching, study selection, data extraction, and synthesis by Tricco et al [31]. A PRISMA (Preferred Reporting Items for Systematic Reviews and Meta-Analyses) flow diagram of our review process is shown in Figure 1 [32]. A PRISMA checklist is provided in Multimedia Appendix 1 [32].

### Objective and Questions

The objective of this rapid umbrella review was to examine the use of AI quality standards based on a sample of published health care AI systematic reviews. Specifically, our questions were as follows:

1. What AI quality standards have been applied to evaluate the quality of health care AI studies?
2. What key quality standard–related issues are noted in these reviews?
3. What guidance can be offered to improve the quality of health care AI studies through the incorporation of AI quality standards?

### Search Strategy

Our search strategy focused on the MEDLINE database supplemented with Google Scholar. Our search terms consisted of “artificial intelligence” or “AI,” “health,” and “systematic review” mentioned in the abstract (refer to Multimedia Appendix 2 for the search strings used). We used the .TW search field tag as it searches on title and abstract as well as fields such as abstract, Medical Subject Heading terms, and Medical Subject Heading subheadings. Our rationale to limit the search to MEDLINE with simple terms was to keep the process manageable, recognizing the huge volume of health care AI–related literature reviews that have appeared in the last few years, especially on COVID-19. One author conducted the MEDLINE and Google Scholar searches with assistance from an academic librarian. For Google Scholar, we restricted the search to the first 100 citations returned.

### Inclusion Criteria

We considered all English-language systematic review articles published over a 36-month period from January 1, 2020, to December 31, 2022. The review could be any type of systematic review, meta-analysis, narrative review, qualitative review, scoping review, meta-synthesis, realist review, or umbrella review as defined in the review typology by MacEntee [29]. The overarching inclusion criteria were AI and health as the focus. To be considered for inclusion, the review articles must meet the following criteria:

1. Each original study in the review is described, where an AI approach in the form of a model, method, algorithm, technique, or intervention is proposed, designed, implemented, or evaluated within a health care context to address a particular health care problem or topic area.
2. We define AI as a simulation of the approximation of human intelligence in machines that comprises learning, reasoning, and logic [33]. In that approximation, AI has different levels of adaptivity and autonomy. Weak AI requires supervision or reinforced learning with human intervention to adapt to the environment, with low autonomous interaction. Strong AI is highly adaptive and highly autonomous via unsupervised learning, with no human intervention.
3. We looked through all the articles, and our health care context categorization was informed by the stated settings (eg, hospital) and purpose (eg, diagnosis) mentioned in the included reviews.
4. The review can include all types of AI approaches, such as ML, NLP, speech recognition, prediction models, neural networks, intelligent robotics, and AI-assisted and automated medical devices.
5. The review must contain sufficient detail on the original AI studies, covering their objectives, contexts, study designs, AI approaches, measures, outcomes, and reference sources.

### Exclusion Criteria

We excluded articles if any one of the following applied:

1. Review articles published before January 1, 2020; not accessible in web-based format; or containing only an abstract
2. Review articles in languages other than English
3. Earlier versions of the review article with the same title or topic by the same authors
4. Context not health care–related, such as electronic commerce or smart manufacturing
5. The AI studies not containing sufficient detail on their purpose, features, or reference sources
6. Studies including multiple forms of digital health technologies besides AI, such as telehealth, personal health records, or communication tools

### Review Article Selection

One author conducted the literature searches and retrieved the citations after eliminating duplicates. The author then screened the citation titles and abstracts against the inclusion and exclusion criteria. Those that met the inclusion criteria were retrieved for full-text review independently by 2 other authors. Any disagreements in final article selection were resolved through consensus between the 2 authors or with a third author. The excluded articles and the reasons for their exclusion were logged.

### Quality Appraisal

In total, 2 authors applied the JBI critical appraisal checklist independently to appraise the quality of the selected reviews [30]. The checklist has 11 questions that allow for *yes*, *no*, *unclear*, or *not applicable* as the response. The questions cover the areas of review question, inclusion criteria, search strategy and sources, appraisal criteria used, use of multiple reviewers, methods of minimizing data extraction errors and combining studies, publication bias, and recommendations supported by data. The reviews were ranked as high, medium, and low quality based on their JBI critical appraisal score (≥0.75 was high quality, ≥0.5 and <0.75 was medium quality, and <0.5 was low quality). All low-quality reviews were excluded from the final synthesis.

### Data Extraction

One author extracted data from selected review articles using a predefined template. A second author validated all the articles for correctness and completeness. As this review was focused on AI quality standards, we extracted data that were relevant to this topic. We created a spreadsheet template with the following data fields to guide data extraction:

1. Author, year, and reference: first author last name, publication year, and reference number
2. URL: the URL where the review article can be found
3. Objective or topic: objective or topic being addressed by the review article
4. Type: type of review reported (eg, systematic review, meta-analysis, or scoping review)
5. Sources: bibliographic databases used to find the primary studies reported in the review article
6. Years: period of the primary studies covered by the review article
7. Studies: total number of primary studies included in the review article
8. Countries: countries where the studies were conducted
9. Settings: study settings reported in the primary studies of the review article
10. Participants: number and types of individuals being studied as reported in the review article
11. AI approaches: the type of AI model, method, algorithm, technique, tool, or intervention described in the review article
12. Life cycle and design: the stage or design of the AI study in the AI life cycle in the primary studies being reported, such as requirements, design, implementation, monitoring, experimental, observational, training-test-validation, or controlled trial
13. Appraisal: quality assessment of the primary studies using predefined criteria (eg, risk of bias)
14. Rating: quality assessment results of the primary studies reported in the review article
15. Measures: performance criteria reported in the review article (eg, mortality, accuracy, and resource use)
16. Analysis: methods used to summarize the primary study results (eg, narrative or quantitative)
17. Results: aggregate findings from the primary studies in the review article
18. Standards: name of the quality standards mentioned in the review article
19. Comments: issues mentioned in the review article relevant to our synthesis

### Removing Duplicate AI Studies

We identified all unique AI studies across the selected reviews after eliminating duplicates that appeared in them. We retrieved full-text articles for every tenth of these unique studies and searched for mention of AI quality standard–related terms in them. This was to ensure that all relevant AI quality standards were accounted for even if the reviews did not mention them.

### Analysis and Synthesis

Our analysis was based on a set of recent publications on health care AI standards. These include (1) the AI life cycle step-by-step approach by van de Sande et al [23] with a list of AI quality standards as benchmarks, (2) the reporting guidelines by Shelmerdine et al [15] with specific standards for different AI-based clinical studies, (3) the international standards for evaluating health care AI by Wenzel and Wiegand [26], and (4) the broader requirements for trustworthy health care AI across the entire life cycle stages by the National Academy of Medicine (NAM) [8] and the European Union Commission (EUC) [34]. As part of the synthesis, we created a conceptual organizing scheme drawing on published literature on AI domains and approaches to visualize their relationships (via a Euler diagram) [35]. All analyses and syntheses were conducted by one author and then validated by another to resolve differences.

For the analysis, we (1) extracted key characteristics of the selected reviews based on our predefined template; (2) summarized the AI approaches, life cycle stages, and quality standards mentioned in the reviews; (3) extracted any additional AI quality standards mentioned in the 10% sample of unique AI studies from the selected reviews; and (4) identified AI quality standard–related issues reported.

For the synthesis, we (1) mapped the AI approaches to our conceptual organizing scheme, visualized their relationships with the AI domains and health topics found, and described the challenges in harmonizing these terms; (2) established key themes from the AI quality standard issues identified and mapped them to the NAM and EUC frameworks [8,34]; and (3) created a summary list of the AI quality standards found and mapped them to the life cycle phases by van de Sande et al [23].

Drawing on these findings, we proposed a set of guidelines that can enhance the quality of future health care AI studies and described its practice, policy, and research implications. Finally, we identified the limitations of this rapid umbrella review as caveats for the readers to consider. As health care, AI, and standards are replete with industry terminologies, we used the acronyms where they are mentioned in the paper and compiled an alphabetical acronym list with their spelled-out form at the end of the paper.

## Results

### Summary of Included Reviews

We found 69 health care AI systematic review articles published between 2020 and 2022, of which 35 (51%) met the inclusion criteria. The included articles covered different review types, topics, settings, numbers of studies, designs, participants, AI approaches, and performance measures (refer to Multimedia Appendix 3 [36-68] for the review characteristics). We excluded the remaining 49% (34/69) of the articles because they (1) covered multiple technologies (eg, telehealth), (2) had insufficient detail, (3) were not specific to health care, or (4) were not in English (refer to Multimedia Appendix 4 for the excluded reviews and reasons). The quality of these reviews ranged from JBI critical appraisal scores of 1.0 to 0.36, with 49% (17/35) rated as high quality, 40% (14/35) rated as moderate quality, and 6% (2/35) rated as poor quality (Multimedia Appendix 5 [36-68]). A total of 6% (2/35) of the reviews were excluded for their low JBI scores [69,70], leaving a sample of 33 reviews for the final synthesis.

Regarding review types, most (23/33, 70%) were systematic reviews [37-40,45-51,53-57,59-64,66,67], with the remaining being scoping reviews [36,41-44,52,58,65,68]. Only 3% (1/33) of the reviews were meta-analyses [38], and another was a rapid review [61]. Regarding health topics, the reviews spanned a wide range of specific health conditions, disciplines, areas, and practices. Examples of conditions were COVID-19 [36,37,49,51,56,62,66], mental health [48,65,68], infection [50,59,66], melanoma [57], and hypoglycemia [67]. Examples of disciplines were public health [36,37,56,66], nursing [42,43,61], rehabilitation [52,64], and dentistry [55,63]. Areas included mobile health and wearables [41,52,54,65], surveillance and remote monitoring [51,61,66], robotic surgeries [47], and biobanks [39]. Practices included diagnosis [37,47,49,58,59,62], prevention [47], prediction [36,38,49,50,57], disease management [41,46,47,58], and administration [42]. Regarding settings, less than half (12/33, 36%) were explicit in their health care settings, which included multiple sources [36,42,43,50,54,61], hospitals [45,49], communities [44,51,58], and social media groups [48]. The number of included studies ranged from 794 on COVID-19 [49] to 8 on hypoglycemia [67]. Regarding designs, most were performance assessment studies using secondary data sources such as intensive care unit [38], imaging [37,62,63], and biobank [39] databases. Regarding participants, they included patients, health care providers, educators, students, simulated cases, and those who use social media. Less than one-third of the reviews (8/33, 24%) mentioned sample sizes, which ranged from 11 adults [44] to 1,547,677 electronic medical records [40] (refer to Multimedia Appendix 3 for details).

Regarding AI approaches, there were >60 types of AI models, methods, algorithms, tools, and techniques mentioned in varying levels of detail across the broad AI domains of computer science, data science with and without NLP, and robotics. The main AI approaches were ML and deep learning (DL), with support vector machine, convolutional neural network, neural network, logistic regression, and random forest being mentioned the most (refer to the next section for details). The performance measures covered a wide range of metrics, such as diagnostic and prognostic accuracies (eg, sensitivity, specificity, accuracy, and area under the curve) [37-40,46-48,53,57,59,63,67], resource use (eg, whether an intensive care unit stay was necessary, length of stay, and cost) [37,58,62], and clinical outcomes (eg, COVID-19 severity, mortality, and behavior change) [36,37,49,56,62,65]. A few reviews (6/33, 18%) focused on the extent of the socioethical guidelines addressed [44,51,55,58,66,68]. Regarding life cycle stages, different schemes were applied, including preprocessing and classification [48,57], data preparation-preprocessing [37,38], different stages of adoption (eg, knowledge, persuasion, decision making, implementation) [44], conceptual research [42], model development [36,37,40,42,45,46,50-56,58-64,66,67], design [43], training and testing [38,42,45,50-53,58,61-64], validation [36-38,40,45,46,50,51,53,55,56,58-64,67], pilot trials [65], public engagement [68], implementation [42,44,60-62,66,68], confirmation [44], and evaluation [42,43,53,60-62,65] (refer to Multimedia Appendix 3 for details). It is worth noting that the period covered for our review did not include any studies on large language models (LLMs). LLM studies became more prevalent in the literature in the period just after our review.

### Use of Quality Standards in Health Care AI Studies

**Figure 2:**
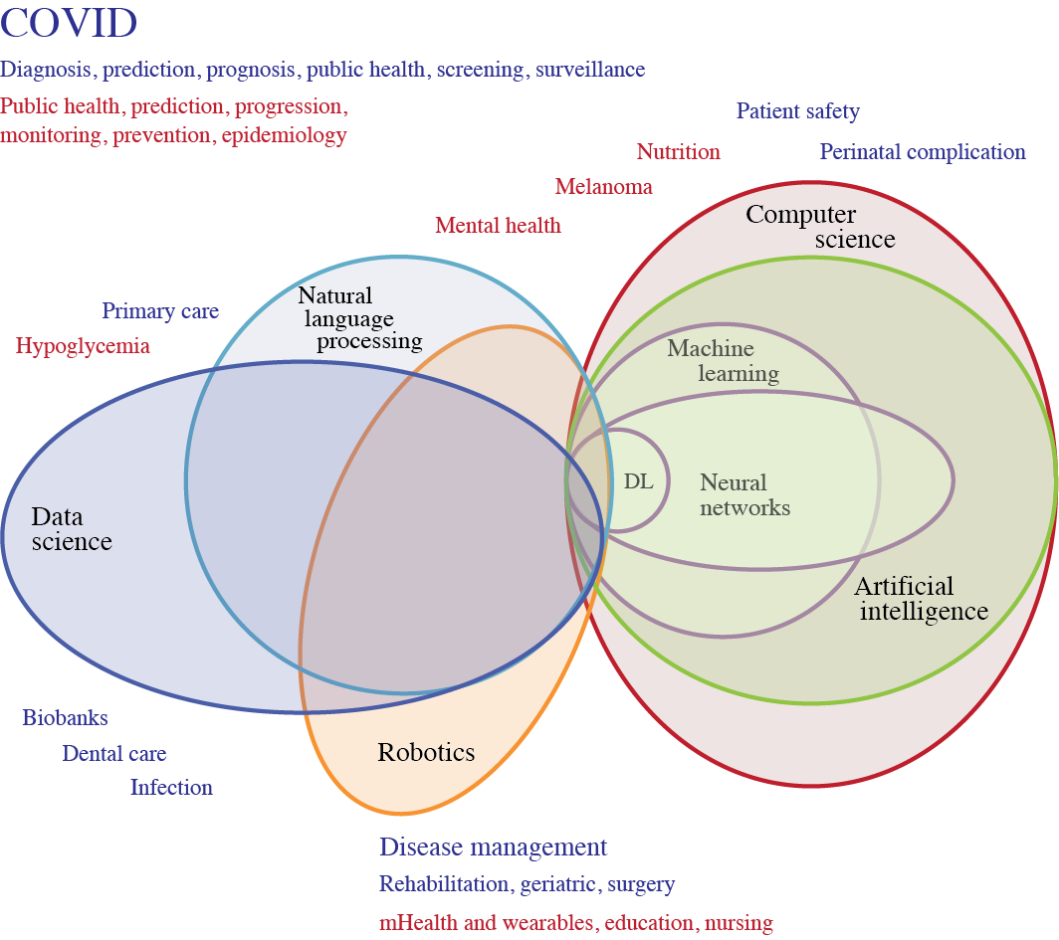
Euler diagram showing the overlapping artificial intelligence (AI) domains and health topics. Health topics in red are from reviews with no mention of specific AI quality standards. Health-related subjects in blue are from reviews with mention of AI quality standards. DL: deep learning; mHealth: mobile health.

To make sense of the different AI approaches mentioned, we used a Euler diagram [71] as a conceptual organizing scheme to visualize their relationships with AI domains and health topics (Figure 2 [36,41-43,47,48,51-54,56-58,60,62,65,67]). The Euler diagram shows that AI broadly comprised approaches in the domains of computer science, data science with and without NLP, and robotics that could be overlapping. The main AI approaches were ML and DL, with DL being a more advanced form of ML through the use of artificial neural networks [33]. The diagram also shows that AI can exist without ML and DL (eg, decision trees and expert systems). There are also outliers in these domains with borderline AI-like approaches mostly intended to enhance human-computer interactions, such as social robotics [42,43], robotic-assisted surgery [47], and exoskeletons [54]. The health topics in our reviews spanned the AI domains, with most falling within data science with or without NLP. This was followed by computer science mostly for communication or database and other functional support and robotics for enhanced social interactions that may or may not be AI driven. There were borderline AI approaches such as programmed social robotics [42,43] or AI-enhanced social robots [54]. These approaches focus on AI enabled social robotic programming and did not use ML or DL. Borderline AI approaches also included virtual reality [60] and wearable sensors [65,66,68].

Regarding AI life cycle stages, we harmonized the different terms used in the original studies by mapping them to the 5 life cycle phases by van de Sande et al [23]: 0 (preparation), I (model development), II (performance assessment), III (clinical testing), and IV (implementation). Most AI studies in the reviews mapped to the first 3 life cycle phases by van de Sande et al [23]. These studies would typically describe the development and performance of the AI approach on a given health topic in a specific domain and setting, including their validation, sometimes done using external data sets [36,38]. A small number of reviews reported AI studies that were at the clinical testing phase [60,61,66,68]. A total of 7 studies were described as being in the implementation phase [66,68]. On the basis of the descriptions provided, few of the AI approaches in the studies in the AI reviews had been adopted for routine use in clinical settings [66,68] with quantifiable improvements in health outcomes (refer to Multimedia Appendix 6 [36-68] for details).

Regarding AI quality standards, only 39% (13/33) of the reviews applied specific AI quality standards in their results [37-40,45,46,50,54,58,59,61,63,66], and 12% (4/33) mentioned the need for standards [55,63,68]. These included the Prediction Model Risk of Bias Assessment Tool [37,38,58,59], Newcastle-Ottawa Scale [39,50], Critical Appraisal and Data Extraction for Systematic Reviews of Prediction Modeling Studies [38,59], Transparent Reporting of a Multivariable Prediction Model for Individual Prognosis or Diagnosis–Machine Learning Extension [50], levels of evidence [61], Critical Appraisal Skills Program Clinical Prediction Rule Checklist [40], Mixed Methods Appraisal Tool [66], and CONSORT-AI [54]. Another review applied 7 design justice principles as the criteria to appraise the quality of their AI studies [68]. There were also broader-level standards mentioned. These included the European Union ethical guidelines for trustworthy AI [44]; international AI standards from the International Organization for Standardization (ISO); and AI policy guidelines from the United States, Russia, and China [46] (refer to Multimedia Appendix 6 for details). We updated the Euler diagram (Figure 2 [36,41-43,47,48,51-54,56-58,60,62,65,67]) to show in red the health topics in reviews with no mention of specific AI standards.

Of the 178 unique original AI studies from the selected reviews that were examined, only 25 (14%) mentioned the use of or need for specific AI quality standards (refer to Multimedia Appendix 7 [36-68] for details). They were of six types: (1) reporting—COREQ (Consolidated Criteria for Reporting Qualitative Research), Strengthening the Reporting of Observational Studies in Epidemiology, Standards for Reporting Diagnostic Accuracy Studies, PRISMA, and EQUATOR; (2) data—Unified Medical Language System, Food and Drug Administration (FDA) Adverse Event Reporting System, MedEx, RxNorm, Medical Dictionary for Regulatory Activities, and PCORnet; (3) technical—ISO-12207, FDA Software as a Medical Device, EU-Scholarly Publishing and Academic Resources Coalition, Sensor Web Enablement, Open Geospatial Consortium, Sensor Observation Service, and the American Medical Association AI recommendations; (4) robotics—ISO-13482 and ISO and TC-299; (5) ethics—Helsinki Declaration and European Union AI Watch; and (6) regulations—Health Insurance Portability and Accountability Act (HIPAA) and World Health Organization World Economic Forum. These standards were added to the list of AI quality standards mentioned by review in Multimedia Appendix 6.

A summary of the harmonized AI topics, approaches, domains, the life cycle phases by van de Sande et al [23], and quality standards derived from our 33 reviews and 10% of unique studies within them is shown in Table 1.

**Table 1:** Summary of artificial intelligence (AI) approaches, domains, life cycle phases, and quality standards in the reviews.

Review, year	Topics	Approaches; examples only (source from original review)	Domains^a^	Life cycle phases	Quality standards^b^
Abd-Alrazaq et al [36], 2020	Public health, risk prediction, and COVID-19	CNN^c^, SVM^d^, RF^e^, DT^f^, and LoR^g^	Data science with NLP^h^	Phase 0, I, and II^i^	Not mentioned; *Helsinki declaration*^*b*^
Adamidi et al [37], 2021	Public health, COVID-19, screening, diagnosis, and prognosis	AB^j^, ARMED^k^, BE^l^, BNB^m^, and CNN	Data science	Phase 0, I, and II	PROBAST^n^, *TRIPOD*^*b,*^^o^ *FDA-SaMD*^*b,*^^p^, and *STROBE*^*b,*^^q^
Barboi et al [38], 2022	Prediction, mortality, and ICU^r^	ANN-ELM^s^, DT, ELM^t^, ensemble LSTM^u^, and ESICULA^v^	Data science with NLP	Phase 0, I, and II	CHARMS^w^ and PROBAST
Battineni et al [39], 2022	Biobanks	CNN and SFCN^x^	Data science and computer science	Phase 0 and I, maybe II	NOS^y^
Bertini et al [40], 2022	Perinatal and complications	AB, ANN^z^, DT, EN^aa^, and GAM^ab^	Computer science	Phase 0, I, and II with need for phase-III clinical testing	CASP^ac^
Bhatt et al [41], 2022	mHealth^ad^ and disease management	DL^ae^ and FL^af^	Data science	Phase 0 and I	Not mentioned
Buchanan et al [42], 2020	Administration, clinical practice, policy, and research	ML^ag^, SAR^ah^, CDSS^ai^, and chatbots	Data science and robotics (x)	Phase 0, implied need for nurses to be involved in all phases	Not mentioned; *COREQ*^aj,b^, *ISO-13482*^*ak,b*^, *EU-SPARC*^al,b^, and *ISO and TC299*, *AM*^am,b^
Buchanan et al [43], 2021	Education	ML, virtual avatar applications, chatbots, wearable armband with ML, and predictive analysis	Data science and robotics (x)	Phase 0, implied need for nurses to be part of co-design at all stages	Not mentioned
Chew and Achananuparp [44], 2022	General	Chatbots, weak AI, image recognition, AI diagnosis, and NLP	Data science with NLP and robotics	Implied phase 0	Not mentioned
Choudhury et al [45], 2020	Patient safety outcomes	ANN, BICMM^an^, BNC^ao^, C4.5^ap^, and CPH^aq^	Data science	Phase 0 and I	ISO and IEC^ar^ 23053, ISO 22100-5, Laskai, NIST^as^, and OECD-AI^at^
Choudhury and Asan [46], 2020	Geriatrics and disease management	AUC^au^, AI, BCP-NN^av^, BCPNN^aw^, and BNM^ax^	Data science with NLP	Phase 0 and I	TRIPOD, TRIPOD-ML^ay^ (cited but not used in AI studies; cited NIST standards), *FAERS*^az,b^, *MedEx*^*b*^, *RxNorm*^*b*^, *MedDRA*^ba,b^, *PCORnet*^*b*^, and *MADE1.0*^bb,b^
Eldaly et al [47], 2022	Lymphedema, prevention, diagnosis, and disease management	ANN, ANFIS^bc^, chatbots, DT, and EML^bd^	Data science and robotics	Phase 0 and I	Not mentioned
Le Glaz et al [48], 2021	Mental health	C4.5, CRA^be^, CUI^bf^, DT, and KM^bg^	Data science with NLP and computer science	Phase 0 and I	Not mentioned; *UMLS*^bh,b^
Guo et al [49], 2021	Prediction, diagnosis, prognosis, and COVID-19	3DQI^bi^, ACNN^bj^, AB, AI, and ANN	Data science	Phase 0 and I	Not mentioned
Hassan et al [50], 2021	Prediction and sepsis	InSight, LASSO^bk^, LR, MCRM^bl^, and MLR^bm^	Data science and computer science	Phase 0, I, and II	TRIPOD and NOS
Huang et al [51], 2022	Telemedicine, monitoring, and COVID-19	CNN-TF^bn^, IRRCNN^bo^, IoT^bp^-based wearable monitoring device, NVHODL^bq^, and SVM	Data science	Phase 0 and I	Not mentioned
Kaelin et al [52], 2021	Pediatric and rehabilitation	NLP, ML, computer vision, and robotics	Data science, computer science, and robotics	Phase 0 and I	Not mentioned; *HIPAA*^br,b^ and *COREQ*^*b*^
Kirk et al [53], 2021	Nutrition	BC^bs^, CNN, DL, DT, and EM^bt^	Data science and computer science	Phase 0, I, and II; the mapping to these phases is questionable	Not mentioned (not even PRISMA^bu^)
Loveys et al [54], 2022	Geriatrics and interventions	AI-enhanced robots, social robots, environmental sensors, and wearable sensors	Data science, computer science, and robotics (x)	Phase 0 and I	Revised Cochrane risk-of-bias tool for RCTs^bv^, cluster RCTs, and non-RCTs (ROBINS-I^bw^)
Mörch et al [55], 2021	Dentistry and ethics	DL, DSP^bx^, ML, and NN^by^	Data science	Phase 0 and I	Mentioned need for SPIRIT^bz^ and TRIPOD, used 2018 Montreal Declaration as AI ethical framework
Payedimarri et al [56], 2021	Public health, interventions, and COVID-19	ABS^ca^, LiR^cb^, NN, and TOPSIS^cc^	Data science	Phase 0 and I	Not mentioned
Popescu et al [57], 2022	Cancer and prediction melanoma	ABC^cd^, autoencoder, CNN, combined networks, and DCNN^ce^	Data science and computer science	Phase 0 and I	Not mentioned
Abbasgholizadeh Rahimi et al [58], 2021	Primary care, diagnosis, and disease management	AL^cf^, AR^cg^, BN^ch^, COBWEB^ci^, and CH^cj^	Data science with NLP	Phase 0 and I	PROBAST
Sahu et al [59], 2022	Detection, neonatal, and sepsis	AR-HMM^ck^, LiR, LoR, MLoR^cl^, and NN	Data science	Phase 0 and I	CHARMS and PROBAST
Sapci and Sapci [60], 2020	Education	AI, DL, ITS^cm^, ML, and NLP	Data Science with NLP and computer science (x)	Phase 0 and I for AI education and training	Not mentioned; *AMA* *–augmented intelligence*^cn,b^
Seibert et al [61], 2021	Ethics	APS^co^, ML, ES^cp^, hybrid, and NLP	Data science, computer science, and robotics	Phase 0, I, II, and III	Risk of bias; levels of evidence from I to VII and not applicable; *SWE*^cq,b^, *OGC*^cr,b^, *SOS*^cs,b^, *COREQ*^*b*^, and *STROBE*^*b*^
Syeda et al [62], 2021	Epidemiology, diagnosis, disease progression, and COVID-19	ANN, BiGAN^ct^, CNN, DT, and DL	Data science	Phase 0, I, and II	Not mentioned
Talpur et al [63], 2022	Dentistry and caries	ADA-NN^cu^, ANN, CNN, F-CNN^cv^, and FFBP-ANN^cw^	Data science	Phase 0, I, and II	Risk-of-bias assessment, no other standards mentioned
Vélez-Guerrero et al [64], 2021	Rehabilitation	AL, ANN, AFM^cx^, ATC^cy^, and AFC^cz^	Data science, computer science, and robotics	Phase 0, I, and II	Need standardized protocol for clinical evaluation, FDA^da^ regulatory standards
Welch et al [65], 2022	Diagnosis, pediatrics, and psychiatry	ML and wearable biosensors	Data science with NLP and computer science (x)	Phase 0, I, and II	Not mentioned
Zhao et al [66], 2021	Public health, surveillance, and COVID-19	AI, AR, ML, physiological monitoring, and sensory technologies	Data science (x)	Phase 0, I, II, III, and IV; the mapping to these phases is questionable	MMAT^db^ for study quality, Asadi framework for ethics, and *STARD*^dc,b^
Zheng et al [67], 2022	Detection and hypoglycemia	ML or rule-based NLP	Data science with NLP (rule-based)	Phase 0, I, and II	Not mentioned
Zidaru et al [68], 2020	Mental health	ML, NLP, sentiment analysis, VR^dd^, and wearable biosensors	Data science (x)	Phase 0, I, II, III, and IV; the mapping to these phases is questionable	Need standards for evaluating safety, outcomes, acceptability, explainability, and inclusive design; *EU AI Watch*^de,b^, *FDA-SaMD*^*b*^, *EQUATOR*^df,b^*, WHO-WEF governance*^dg,b^, *CCC AI road map*^dh,b^, and *ISO, IEC, or IEEE-12207*^di,b^

^a^Borderline AI approaches in the AI domains are identified with *(x)*.

^b^Italicized entries are AI quality standards mentioned only in the original studies in the reviews.

^c^CNN: convolutional neural network.

^d^SVM: support vector machine.

^e^RF: random forest.

^f^DT: decision tree.

^g^LoR: logistic regression.

^h^NLP: natural language processing.

^i^Phase 0: preparation before model development; phase I: AI model development; phase II: assessment of AI performance and reliability; phase III: clinical testing of AI; and phase IV: implementing and governing AI.

^j^AB: adaptive boosting or adaboost.

^k^ARMED: attribute reduction with multi-objective decomposition ensemble optimizer.

^l^BE: boost ensembling.

^m^BNB: Bernoulli naïve Bayes.

^n^PROBAST: Prediction Model Risk of Bias Assessment Tool.

^o^TRIPOD: Transparent Reporting of a Multivariable Prediction Model for Individual Prognosis or Diagnosis.

^p^FDA-SaMD: Food and Drug Administration–Software as a Medical Device.

^q^STROBE: Strengthening the Reporting of Observational Studies in Epidemiology.

^r^ICU: intensive care unit.

^s^ANN-ELM: artificial neural network extreme learning machine.

^t^ELM: ensemble machine learning.

^u^LSTM: long short-term memory.

^v^ESICULA: super intensive care unit learner algorithm.

^w^CHARMS: Checklist for Critical Appraisal and Data Extraction for Systematic Reviews of Prediction Modeling Studies.

^x^SFCN: sparse fully convolutional network.

^y^NOS: Newcastle-Ottawa scale.

^z^ANN: artificial neural network.

^aa^EN: elastic net.

^ab^GAM: generalized additive model.

^ac^CASP: Critical Appraisal Skills Programme.

^ad^mHealth: mobile health.

^ae^DL: deep learning.

^af^FL: federated learning.

^ag^ML: machine learning.

^ah^SAR: socially assistive robot.

^ai^CDSS: clinical decision support system.

^aj^COREQ: Consolidated Criteria for Reporting Qualitative Research.

^ak^ISO: International Organization for Standardization.

^al^EU-SPARC: Scholarly Publishing and Academic Resources Coalition Europe.

^am^AMS: Associated Medical Services.

^an^BICMM: Bayesian independent component mixture model.

^ao^BNC: Bayesian network classifier.

^ap^C4.5: a named algorithm for creating decision trees.

^aq^CPH: Cox proportional hazard regression.

^ar^IEC: international electrotechnical commission.

^as^NIST: National Institute of Standards and Technology.

^at^OECD-AI: Organisation for Economic Co-operation and Development–artificial intelligence.

^au^AUC: area under the curve.

^av^BCP-NN: Bayesian classifier based on propagation neural network.

^aw^BCPNN: Bayesian confidence propagation neural network.

^ax^BNM: Bayesian network model.

^ay^TRIPOD-ML: Transparent Reporting of a Multivariable Prediction Model for Individual Prognosis or Diagnosis–Machine Learning.

^az^FAERS: Food and Drug Administration Adverse Event Reporting System.

^ba^MedDRA: Medical Dictionary for Regulatory Activities.

^bb^MADE1.0: Medical Artificial Intelligence Data Set for Electronic Health Records 1.0.

^bc^ANFIS: adaptive neuro fuzzy inference system.

^bd^EML: ensemble machine learning.

^be^cTAKES: clinical Text Analysis and Knowledge Extraction System.

^bf^CUI: concept unique identifier.

^bg^KM: k-means clustering.

^bh^UMLS: Unified Medical Language System.

^bi^3DQI: 3D quantitative imaging.

^bj^ACNN: attention-based convolutional neural network.

^bk^LASSO: least absolute shrinkage and selection operator.

^bl^MCRM: multivariable Cox regression model.

^bm^MLR: multivariate linear regression.

^bn^CNN-TF: convolutional neural network using Tensorflow.

^bo^IRRCN: inception residual recurrent convolutional neural network.

^bp^IoT: internet of things.

^bq^NVHDOL: notal vision home optical-based deep learning.

^br^HIPAA: Health Insurance Portability and Accountability Act.

^bs^BC: Bayesian classifier.

^bt^EM: ensemble method.

^bu^PRISMA: Preferred Reporting Items for Systematic Reviews and Meta-Analyses.

^bv^RCT: randomized controlled trial.

^bw^ROBINS-I: Risk of Bias in Non-Randomised Studies of Interventions.

^bx^DSP: deep supervised learning.

^by^NN: neural network.

^bz^SPIRIT: Standard Protocol Items: Recommendations for Interventional Trials.

^ca^ABS: agent based simulation.

^cb^LiR: linear regression.

^cc^TOPSIS: technique for order of preference by similarity to ideal solution.

^cd^ABC: artificial bee colony.

^ce^DCNN: deep convolutional neural network.

^cf^AL: abductive learning.

^cg^AR: automated reasoning.

^ch^BN: Bayesian network.

^ci^COBWEB: a conceptual clustering algorithm.

^cj^CH: computer heuristic.

^ck^AR-HMM: auto-regressive hidden Markov model.

^cl^MLoR: multivariate logistic regression.

^cm^ITS: intelligent tutoring system.

^cn^AMA: American Medical Association.

^co^APS: automated planning and scheduling.

^cp^ES: expert system.

^cq^SWE: software engineering.

^cr^OGC: open geospatial consortium standard.

^cs^SOS: start of sequence.

^ct^BiGAN: bidirectional generative adversarial network.

^cu^ADA-NN: adaptive dragonfly algorithms with neural network.

^cv^F-CNN: fully convolutional neural network.

^cw^FFBP-ANN: feed-forward backpropagation artificial neural network.

^cx^AFM: adaptive finite state machine.

^cy^ATC: anatomical therapeutic chemical.

^cz^AFC: active force control.

^da^FDA: Food and Drug Administration.

^db^MMAT: Mixed Methods Appraisal Tool.

^dc^STARD: Standards for Reporting of Diagnostic Accuracy Study.

^dd^VR: virtual reality.

^de^EU: European Union.

^df^EQUATOR: Enhancing the Quality and Transparency of Health Research.

^dg^WHO-WEF: World Health Organization World Economic Forum.

^dh^CCC: concordance correlation coefficient.

^di^IEEE: Institute of Electrical and Electronics Engineers.

There were also other AI quality standards not mentioned in the reviews or their unique studies. They included guidelines such as the do no harm road map, Factor Analysis of Information Risk, HIPAA, and the FDA regulatory framework mentioned by van de Sande et al [23]; AI clinical study reporting guidelines such as Clinical Artificial Intelligence Modeling and Minimum Information About Clinical Artificial Intelligence Modeling mentioned by Shelmerdine et al [15]; and the international technical AI standards such as ISO and International Electrotechnical Commission 22989, 23053, 23894, 24027, 24028, 24029, and 24030 mentioned by Wenzel and Wiegand [26].

With these additional findings, we updated the original table of AI standards in the study by van de Sande et al [23] showing crucial steps and key documents by life cycle phase (Table 2).

**Table 2:** Use of health care standards in the reviews mapped to the life cycle phases by van de Sande et al [23].

			Standards and corresponding reviews^a^
**Life cycle phase 0: preparation before AI^b^ model development**			
	Define the problem and engage stakeholders	Do no harm road map (Wiens)	
	Search for and evaluate available models	FDA^c^ devices (Benjamens) ÉCLAIR^d^ (Omoumi)	
	Identify, collect data, and account for bias	FHIR^e^ (Mandel) FAIR^f^ (Wilkinson) Validation (Riley) PROBAST^g^ (Moons and Wolff): *Adamidi et al* [37]^a^, Barboi et al [38], Abbasgholizadeh Rahimi et al [58], and Sahu et al [59]	
	Handle privacy	HIPAA^h^ (OOTA^i^): Battineni et al [39] GDPR^j^ (EU^k^)	
	Ethical principles, frameworks, and guidelines	WMA^l^ (Helsinki declaration): *Abd-Alrazaq et al* [36]^a^ Ethics for mental health technology (WEF^m^): Zidaru et al [68] Digital disease technology detection (SORMAS^n^): *Zhao et al* [13]^a^ Asadi framework of ethics (Asadi): *Zhao et al* [15]^a^ Ethical guidelines (EU): Chew and Achananuparp [44] Ethical principles and framework (Montreal): Mörch et al [55] Ethics and governance of AI health (WHO^o^): Zidaru et al [68]	
**Life cycle phase I: AI model development**			
	Check applicable regulations	Proposed regulatory framework (FDA) Harmonized rules on AI (EU)	
	Prepare data	Preprocessing data (Ferrao)	
	Train and validate	ML^p^ cardiac imaging (Juarez-Orozco)	
	Evaluate performance and report results	AI guide (Park and Han): Mörch et al [55] TRIPOD^q^ (Collins): Adamidi et al [37] TRIPOD-ML^r^ (Collins): Hassan et al [50] and Mörch et al [55] CLAIM^s^ (Mongan): Shelmerdine et al [15]^t^ CHARMS^u^ (Moons): Barboi et al [38] and Sahu et al [59] PRISMA-DTA^v^ (McInnes): Shelmerdine et al [15]^t^ MI-CLAIM^w^ (Norgeot): Shelmerdine et al [15]^t^ MINIMAR^x^ (Hernandez-Boussard): Shelmerdine et al [15]^t^ NOS^y^ (Lo): Hassan et al [50] and Battineni et al [39] LOE^z^ (Concato): *Seibert et al* [61]^a^ MMAT^aa^ (Hong): *Zhao et al* [66]^a^ Clinical Prediction Rule Checklist (CASP^ab^): Bertini et al [40] STARD^ac^ (checklist): *Zhao et al* [66]^a^ COREQ^ad^ (checklist): *Buchanan et al* [42]^a^, *Kaelin et al* [52]^a^, and *Seibert et al* [61]^a^	
**Life cycle phase II: assessment of AI performance and reliability**			
	Externally validate model or concept	Validation (Ramspek and Riley) Generalizability (Futoma) Risk of bias (?): Talpur et al [63] MADE1.0^ae^ (Dandala): *Choudhury and Asan* [46]^a^	
	Simulate results and prepare for clinical study	DECIDE-AI^af^ (steering group): Shelmerdine et al [15]^t^	
**Life cycle phase III: clinically testing AI**			
	Design and conduct clinical study	SPIRIT-AI^ag^ (Cruz): Mörch et al [55] Explanations (Barda) CONSORT-AI^ah^ (Liu): Loveys et al [54] Revised Cochrane RoB 2^ai^ (Sterne): Loveys et al [54] ROBINS-I^aj^ (RoB 2 for non-RCTs^ak^; Sterne): Loveys et al [54] STROBE^al^ (checklists): *Adamidi et al* [37]^a^	
**Life cycle phase IV: implementing and governing AI**			
	Legal approval	AI-ML^am^ medical devices (Muehlematter)	
	Safely implement model	TAM^an^ (Jauk) ML model facts (Sendak)	
	Model and data governance	FAIR (Wilkinson): *Adamidi et al* [37]^a^ SaMD^ao^ clinical evaluation (FDA): *Adamidi et al* [37]^a^ Quality management system (IMDRF^ap^): *Adamidi et al* [37]^a^	
	Responsible model use	Ethics ambient intelligence (Martinz-Martin)	
**Standards in the reviews mapped to multiple phases**			
	Design justice principles	10 principles—International Design Justice Network: Zidaru et al [68]	
	Study quality	Reporting guidelines (EQUATOR^aq^): Zidaru et al [68]	
	Policy	China—AI governance (Laskai): Choudhury et al [45] Federal engagement plan (NIST^ar^): Choudhury et al [45] Russian AI policy (OECD^as^): Choudhury et al [45] AMA^at^ AI recommendations (link): Sapci and Sapci [60] CCC^au^ AI road map (link): Zidaru et al [68]^a^ EU (AI Watch): Zidaru et al [68]^a^	
	Technical and interoperability	Software life cycle ISO^av^ and IEEE^aw^-12207 (link): Zidaru et al [68]^a^ OGC^ax^: *Seibert et al* [61]^a^ SWE^ay^: *Seibert et al* [61]^a^ SOS^az^: *Seibert et al* [61]^a^ AI concepts and terminology (ISO and IEC^ba^ 22989): *Seibert et al* [61]^a^ Framework for AI systems (ISO and IEC 23053): Wenzel and Wiegand [26]^t^ AI risk management (ISO and IEC 23894): Wenzel and Wiegand [26]^t^ AI bias (ISO and IEC 24027): Wenzel and Wiegand [26]^t^ AI trustworthiness (ISO and IEC 24028): Wenzel and Wiegand [26]^t^ AI robustness (ISO and IEC 24029-1): Wenzel and Wiegand [26]^t^ AI use cases (ISO and IEC 24030): Wenzel and Wiegand [26]^t^ Safety of machinery (ISO 22100-5): *Choudhury et al* [45]^a^	
	Terminology standards	FAERS^bb^: *Choudhury et al* [45]^a^ Medication information extract clinical notes (MedEx): *Choudhury et al* [45]^a^ Medical prescription normalized (RxNorm): *Choudhury et al* [45]^a^ MedDRA^bc^: *Choudhury et al* [45]^a^ Patient-centered clinical research network (PCORnet): *Choudhury et al* [45]^a^ UMLS^bd^: *Choudhury et al* [45]^a^	
**Robotics**			
	Partnership for R&D^be^ and innovation	European robotics partnership (SPARC^bf^): *Buchanan et al* [42]^a^	
	Robotic standardization and safety	Robotics standardization (ISO and TC^bg^ 299): *Buchanan et al* [42]^a^	
	Robotic devices for personal care	Personal care robots (13482: 2014): *Buchanan et al* [42]^a^ Vocabulary (ISO 8373: 2021): *Buchanan et al* [42]^a^	

^a^Italicized references are original studies cited in the reviews, and references denoted with the footnote *t* are those cited in our paper but not present in any of the reviews.

^b^AI: artificial intelligence.

^c^FDA: Food and Drug Administration.

^d^ECLAIR: Evaluate Commercial AI Solutions in Radiology.

^e^FHIR: Fast Healthcare Interoperability Resources.

^f^FAIR: Findability, Accessibility, Interoperability, and Reusability.

^g^PROBAST: Prediction Model Risk of Bias Assessment Tool.

^h^HIPAA: Health Insurance Portability and Accountability Act.

^i^OOTA: Office of The Assistant Secretary.

^j^GDPR: General Data Protection Regulation.

^k^EU: European Union.

^l^WMA: World Medical Association.

^m^WEF: World Economic Forum.

^n^SORMAS: Surveillance, Outbreak Response Management and Analysis System.

^o^WHO: World Health Organization.

^p^ML: machine learning.

^q^TRIPOD: Transparent Reporting of a multivariable prediction model for Individual Prognosis Or Diagnosis.

^r^TRIPOD-ML: Transparent Reporting of a multivariable prediction model for Individual Prognosis Or Diagnosis—Machine Learning.

^s^CLAIM: Checklist for Artificial Intelligence in Medical Imaging.

^t^References denoted with the footnote *t* are those cited in our paper but not present in any of the reviews.

^u^CHARMS: Checklist for Critical Appraisal and Data Extraction for Systematic Reviews of Prediction Modeling Studies.

^v^PRISMA-DTA: Preferred Reporting Items for Systematic Reviews and Meta-Analyses of Diagnostic Test Accuracy.

^w^MI-CLAIM: Minimum Information About Clinical Artificial Intelligence Modeling.

^x^MINIMAR: Minimum Information for Medical AI Reporting.

^y^NOS: Newcastle-Ottawa Scale.

^z^LOE: level of evidence.

^aa^MMAT: Mixed Methods Appraisal Tool.

^ab^CASP: Critical Appraisal Skills Programme.

^ac^STARD: Standards for Reporting of Diagnostic Accuracy Studies.

^ad^COREQ: Consolidated Criteria for Reporting Qualitative Research.

^ae^MADE1.0: Model Agnostic Diagnostic Engine 1.0.

^af^DECIDE-AI: Developmental and Exploratory Clinical Investigations of Decision-Support Systems Driven by Artificial Intelligence.

^ag^SPIRIT-AI: Standard Protocol Items: Recommendations for Interventional Trials–Artificial Intelligence.

^ah^CONSORT-AI: Consolidated Standards of Reporting Trials–Artificial Intelligence.

^ai^RoB 2: Risk of Bias 2.

^aj^ROBINS-I: Risk of Bias in Non-Randomised Studies of Interventions.

^ak^RCT: randomized controlled trial.

^al^STROBE: Strengthening the Reporting of Observational Studies in Epidemiology.

^am^AI-ML: artificial intelligence–machine learning.

^an^TAM: Technology Acceptance Model.

^ao^SaMD: Software as a Medical Device.

^ap^IMDRF: International Medical Device Regulators Forum.

^aq^EQUATOR: Enhancing the Quality and Transparency of Health Research.

^ar^NIST: National Institute of Standards and Technology.

^as^OECD: Organisation for Economic Co-operation and Development.

^at^AMA: American Medical Association.

^au^CCC: Computing Community Consortium.

^av^ISO: International Organization for Standardization.

^aw^IEEE: Institute of Electrical and Electronics Engineers.

^ax^OGC: Open Geospatial Consortium.

^ay^SWE: Sensor Web Enablement.

^az^SOS: Sensor Observation Service.

^ba^IEC: International Electrotechnical Commission.

^bb^FAERS: Food and Drug Administration Adverse Event Reporting System.

^bc^MedDRA: Medical Dictionary for Regulatory Activities.

^bd^UMLS: Unified Medical Language System.

^be^R&D: research and development.

^bf^SPARC: Scholarly Publishing and Academic Resources Coalition.

^bg^TC: technical committee.

### Quality Standard–Related Issues

We extracted a set of AI quality standard–related issues from the 33 reviews and assigned themes based on keywords used in the reviews (Multimedia Appendix 8 [36-68]). In total, we identified 23 issues, with the most frequently mentioned ones being clinical utility and economic benefits (n=10); ethics (n=10); benchmarks for data, model, and performance (n=9); privacy, security, data protection, and access (n=8); and federated learning and integration (n=8). Table 3 shows the quality standard issues by theme from the 33 reviews. To provide a framing and means of conceptualizing the quality-related issues, we did a high-level mapping of the issues to the AI requirements proposed by the NAM [8] and EUC [20]. The mapping was done by 2 of the authors, with the remaining authors validating the results. Final mapping was the result of consensus across the authors (Table 4).

**Table 3:** Summary of quality standard–related issues in the reviews.

Key themes	Quality issues	Reviews
Ethics	Guidelines needed, 10 issues—prudence, equity, privacy and intimacy, democratic participation, solidarity, responsibility, diversity inclusion, well-being, respect for autonomy, and sustainable development (Mörch et al [55]); the individual, organizational and society levels of the ethical framework by Asadi et al (Zhao et al [66])	Abd-Alrazaq et al [36] Bertini et al [40] Buchanan et al [43] Le Glaz et al [48] Loveys et al [54] Mörch et al [55] Abbasgholizadeh Rahimi et al [58] Seibert et al [61] Zhao et al [66] Zidaru et al 69]
Benefits, cost-effective, economic, external and clinical validation, and clinical utility	Need clinical validation and demonstration of economic benefits and clinical utility in real-world settings	Bertini et al [40] Eldaly et al [47] Kirk et al [53] Abbasgholizadeh Rahimi et al [58] Sahu et al [59] Seibert et al [61] Syeda et al [62] Vélez-Guerrero et al [64] Welch et al [65] Zidaru et al [68]
Benchmarks—models, data, and performance	Need standardized and comparable AI^a^ models, parameters, evaluation measures, and gold standards	Barboi et al [38] Bertini et al [40] Choudhury et al [45] Choudhury and Asan [46] Hassan et al [50] Kirk et al [53] Syeda et al [62]
Integration, federated learning, decision fusion, and ability to aggregate results	Need to integrate heterogeneous data from multiple sources and combine multiple classifier outputs into a common decision	Abd-Alrazaq et al [36] Adamidi et al [37] Bhatt et al [41] Guo et al [49] Kirk et al [53] Popescu et al [57] Vélez-Guerrero et al [64] Zheng et al [67]
Privacy, security, open data, access, and protection	Need agreements and processes on privacy, security, access, open data, and data protection	Buchanan et al [43] Choudhury and Asan [46] Le Glaz et al [48] Guo et al [49] Loveys et al [54] Seibert et al [61]
Education, web-based learning, learning experience, and competencies	Need education for the public, patients, students, and providers, including web-based learning and building competencies and as part of formal curricula	Abd-Alrazaq et al [36] Chew and Achananuparp [44] Choudhury and Asan [46] Kirk et al [53] Sapci and Sapci [60] Seibert et al [61]
Explainability	Enhance acceptability, understandability, and interpretability of solutions and ability to convey them to patients	Abd-Alrazaq et al [36] Adamidi et al [37] Bhatt et al [41] Kirk et al [53] Syeda et al [62] Vélez-Guerrero et al [64]
Reporting standards	Standardized reporting of study details to allow for comparison and replication	Abd-Alrazaq et al [36] Barboi et al [38] Bertini et al [40] Choudhury and Asan [46] Abbasgholizadeh Rahimi et al [58] Zheng et al [67]
Transparency	Need openness and being accountable through the entire AI life cycle	Adamidi et al [37] Barboi et al [38] Bhatt et al [41] Chew and Achananuparp [44] Le Glaz et al [48] Huang et al [51]
Trust and trustworthiness	Need ethical guidelines to ensure confidence, truthfulness, and honesty with the design, use, and impact of AI systems	Adamidi et al [37] Bhatt et al [41] Chew and Achananuparp [44] Huang et al [51] Abbasgholizadeh Rahimi et al [58] Zidaru et al [68]
Safety	Need to ensure patient safety from harm	Chew and Achananuparp [44] Choudhury et al [45] Choudhury and Asan [46] Seibert et al [61] Zidaru et al [68]
Bias—SDOH^b^ and assessment	Need to consider sociodemographic variables and adequate sample sizes	Adamidi et al [37] Le Glaz et al [48] Abbasgholizadeh Rahimi et al [58] Syeda et al [62] Vélez-Guerrero et al [64]
Co-design and engagement—user, provider, and public	Meaningful participation at all life cycle stages	Buchanan et al [42] Buchanan et al [43] Huang et al [51] Abbasgholizadeh Rahimi et al [58] Zidaru et al [68]
Technology maturity or feasibility, acceptance, and usability	Need user-friendly and mature AI systems with proven benefits to increase adoption	Chew and Achananuparp [44] Eldaly et al [47] Seibert et al [61]
Regulation and legal	Need legal framework and laws to ensure appropriate safe use and liability protection	Bhatt et al [41] Choudhury and Asan [46] Abbasgholizadeh Rahimi et al [58] Seibert et al [61]
Context and time dependency	Purpose of AI models, health care context, and time lags have mediating effect	Choudhury et al [45] Kaelin et al [52] Payedimarri et al [56]
Data integration and preprocessing	Need to integrate different variables and include multilevel data preprocessing to reduce dimensionality	Guo et al [49] Kirk et al [53]
Design justice, equity, and fairness	Need design justice principles to engage the public and ensure a fair and equitable AI system	Buchanan et al [43] Zidaru et al [68]
Personalized care and targeted interventions	Select the best AI algorithms and outputs to customize care for specific individuals	Battineni et al [39] Kaelin et al [52]
Quality—data and study	Need well-designed studies and quality data to conduct AI studies	Talpur et al [63] Welch et al [65]
Social justice and social implications	Need to balance human caring needs with AI advances, understanding the societal impact of AI interventions	Buchanan et al [43]
Governance	Need governance on the collection, storage, use, and transfer of data; being accountable and transparent with the process	Choudhury et al [45]
Self-adaptability	Need adaptable and flexible AI systems that can improve over time	Vélez-Guerrero et al [64]

^a^AI: artificial intelligence.

^b^SDOH: social determinants of health.

**Table 4:** Quality standard–related issues by theme mapped to the National Academy of Medicine (NAM) and European Union Commission (EUC) requirements.

Key themes	NAM^a^	EUC^b^	Reviews
Ethics	T6-2: ethics and fairness	1—rights, agency, and oversight; 7—minimizing and reporting negative impact	Abd-Alrazaq et al [36] Bertini et al [40] Buchanan et al [43] Le Glaz et al [48] Loveys et al [54] Mörch et al [55] Abbasgholizadeh Rahimi et al [58] Seibert et al [61] Zhao et al [66] Zidaru et al [68]
Benefits, cost-effective, economic, external and clinical validation, and clinical utility	B5-1: accuracy and outcome change; T6-2: cost, revenue and value, and safety and efficacy; and T6-3: improvement and assessments	6—environmentally friendly and sustainable; 7—documenting trade-off and ability to redress	Bertini et al [40] Eldaly et al [47] Kirk et al [53] Abbasgholizadeh Rahimi et al [58] Sahu et al [59] Seibert et al [61] Syeda et al [62] Vélez-Guerrero et al [64] Welch et al [65] Zidaru et al [68]
Benchmarks—models, data, and performance	B5-1: accuracy and outcome change; T6-2: cost, revenue and value, and safety and efficacy; and T6-3: defining success and after-action assessments	2—accuracy, reliability, and reproducibility; 3—data quality and integrity	Barboi et al [38] Bertini et al [40] Choudhury et al [45] Choudhury and Asan [46] Hassan et al [50] Kirk et al [53] Syeda et al [62]
Integration, federated learning, decision fusion, and ability to aggregate results	T6-2: data environment and interoperability	3—data governance; 4—traceability and explainability	Abd-Alrazaq et al [36] Adamidi et al [37] Bhatt et al [41] Guo et al [49] Kirk et al [53] Popescu et al [57] Vélez-Guerrero et al [64] Zheng et al [67]
Privacy, security, open data, access, and protection	T6-2: cybersecurity and privacy	2—resilience; 3—data privacy, protection, and access	Buchanan et al [43] Choudhury and Asan [46] Le Glaz et al [48] Guo et al [49] Loveys et al [54] Seibert et al [61]
Education, web-based learning, learning experience, and competencies	T6-3: education and support	7—minimizing and reporting negative impact	Abd-Alrazaq et al [36] Chew and Achananuparp [44] Choudhury and Asan [46] Kirk et al [53] Sapci and Sapci [60] Seibert et al [61]
Explainability	N/A^c^	4—explainability and communication	Abd-Alrazaq et al [36] Adamidi et al [37] Bhatt et al [41] Kirk et al [53] Syeda et al [62] Vélez-Guerrero et al [64]
Reporting standards	—^d^	3—privacy and data protection; 7—auditability, documenting trade-offs, and ab	Abd-Alrazaq et al [36] Barboi et al [38] Bertini et al [40] Choudhury and Asan [46] Abbasgholizadeh Rahimi et al [58] Zheng et al [67]
Transparency	—	4—traceability and communication	Adamidi et al [37] Barboi et al [38] Bhatt et al [41] Chew and Achananuparp [44] Le Glaz et al [48] Huang et al [51]
Trust and trustworthiness	—	All 7 assessment list items	Adamidi et al [37] Bhatt et al [41] Chew and Achananuparp [44] Huang et al [51] Abbasgholizadeh Rahimi et al [58] Zidaru et al [68]
Safety	T6-2: safety and efficacy	2—resilience and safety	Chew and Achananuparp [44] Choudhury et al [45] Choudhury and Asan [46] Seibert et al [61] Zidaru et al [68]
Bias—SDOH^e^ and assessment	B5-1: accuracy	5—bias avoidance	Adamidi et al [37] Le Glaz et al [48] Abbasgholizadeh Rahimi et al [58] Syeda et al [62] Vélez-Guerrero et al [64]
Co-design and engagement—user, provider, and public	B5-1: target users; T6-2: patient, family, and consumer engagement; and T6-3: stakeholder consensus	1—rights, agency, and oversight; 5—participation	Buchanan et al [42] Buchanan et al [43] Huang et al [51] Abbasgholizadeh Rahimi et al [58] Zidaru et al [68]
Technology maturity or feasibility, acceptance, and usability	—	5—accessibility and universal design	Chew and Achananuparp [44] Eldaly et al [47] Seibert et al [61]
Regulation and legal	T6-2: regulatory issues	7—audibility and minimizing and reporting negative impact	Bhatt et al [41] Choudhury and Asan [46] Abbasgholizadeh Rahimi et al [58] Seibert et al [61]
Context and time dependency	T6-3: problem identification	2—accuracy, reliability, and reproducibility	Choudhury et al [45] Kaelin et al [52] Payedimarri et al [56]
Data integration and preprocessing	—	3—data quality and integrity	Guo et al [49] Kirk et al [53]
Design justice, equity, and fairness	T6-2: ethics and fairness	1—rights, agency, and oversight; 6—environmentally friendly and sustainable	Buchanan et al [43] Zidaru et al [68]
Personalized care and targeted interventions	B5-1: downstream interventions, target users, and capacity to intervene	1—rights, agency, and oversight; 5—bias avoidance, universal design, and accessibility	Battineni et al [39] Kaelin et al [52]
Quality—data and study	—	3—data quality and integrity	Talpur et al [63] Welch et al [65]
Social justice and social implications	B5-1: downstream interventions and desired outcome change	1—rights, agency, and oversight; 6—social impact and society and democracy	Buchanan et al [43]
Governance	T6-2: organizational capabilities, data environment, and personal capacity	3—data quality and integrity; data access	Choudhury et al [45]
Self-adaptability	—	1—oversight	Vélez-Guerrero et al [64]

^a^B5-1: key considerations in model development; T6-2: key considerations for institutional infrastructure and governance; and T6-3: key artificial intelligence tool implementation concepts, considerations, and tasks.

^b^1—human agency and oversight; 2—technical robustness and safety; 3—privacy and data governance; 4—transparency; 5—diversity, nondiscrimination, and fairness; 6—societal and environmental well-being; and 7—accountability.

^c^N/A: not applicable.

^d^Themes not addressed.

^e^SDOH: social determinants of health.

We found that all 23 quality standard issues were covered in the AI frameworks by the NAM and EUC. Both frameworks have a detailed set of guidelines and questions to be considered at different life cycle stages of the health care AI studies. While there was consistency in the mapping of the AI issues to the NAM and EUC frameworks, there were some differences across them. Regarding the NAM, the focus was on key aspects of AI model development, infrastructure and governance, and implementation tasks. Regarding the EUC, the emphasis was on achieving trustworthiness by addressing all 7 interconnected requirements of accountability; human agency and oversight; technical robustness and safety; privacy and data governance; transparency; diversity, nondiscrimination, and fairness; and societal and environmental well-being. The quality standard issues were based on our analysis of the review articles, and our mapping was at times more granular than the issues from the NAM and EUC frameworks. However, our results showed that the 2 frameworks do provide sufficient terminology for quality standard–related issues. By embracing these guidelines, one can enhance the buy-in and adoption of the AI interventions in the health care system.

## Discussion

### Principal Findings

Overall, we found that, despite the growing number of health care AI quality standards in the literature, they are seldom applied in practice, as is shown in a sample of recently published systematic reviews of health care AI studies. Of the reviews that mentioned AI quality standards, most were used to ensure the methodological and reporting quality of the AI studies involved. At the same time, the reviews identified many AI quality standard–related issues, including those broader in nature, such as ethics, regulations, transparency, interoperability, safety, and governance. Examples of broader standards mentioned in a handful of reviews or original studies are the ISO-12207, Unified Medical Language System, HIPAA, FDA Software as a Medical Device, World Health Organization AI governance, and American Medical Association augmented intelligence recommendations. These findings reflect the evolving nature of health care AI, which has not yet reached maturity or been widely adopted. There is a need to apply appropriate AI quality standards to demonstrate the transparency, robustness, and benefits of these AI approaches in different AI domains and health topics while protecting the privacy, safety, and rights of individuals and society from the potential unintended consequences of such innovations.

Another contribution of our study was a conceptual reframing for a systems-based perspective to harmonize health care AI. We did not look at AI studies solely as individual entities but rather as part of a bigger system that includes clinical, organizational, and societal aspects. Our findings complement those of recent publications, such as an FDA paper that advocates for a need to help people understand the broader system of AI in health care, including across different clinical settings [72]. Moving forward, we advocate for AI research that looks at how AI approaches will mature over time. AI approaches evolve through different phases of maturity as they move from development to validation to implementation. Each phase of maturity has different requirements [23] that must be assessed as part of evaluating AI approaches across domains as the number of health care applications rapidly increases [73]. However, comparing AI life cycle maturity across studies was challenging as there were a variety of life cycle terms used across the reviews, making it hard to compare life cycle maturity in and across studies. To address this issue, we provided a mapping of life cycle terms from the original studies but also used the system life cycle phases by van de Sande et al [23] as a common terminology for AI life cycle stages. A significant finding from the mapping was that most AI studies in our selected reviews were still at early stages of maturity (ie, model preparation, development, or validation), with very few studies progressing to later phases of maturity such as clinical testing and implementation. If AI research in health systems is to evolve, we need to move past single-case studies with external data validation to studies that achieve higher levels of life cycle maturity, such as clinical testing and implementation over a variety of routine health care settings (eg, hospitals, clinics, and patient homes and other community settings).

Our findings also highlighted that there are many AI approaches and quality standards used across domains in health care AI studies. To better understand their relationships and the overall construct of the approach, our applied conceptual organizing scheme for harmonized health care characterizes AI studies according to AI domains, approaches, health topics, life cycle phases, and quality standards. The health care AI landscape is complex. The Euler diagram shows multiple AI approaches in one or more AI domains for a given health topic. These domains can overlap, and the AI approaches can be driven by ML, DL, or other types (eg, decision trees, robotics). This complexity is expected to increase as the number of AI approaches and range of applications across all health topics and settings grows over time. For meaningful comparison, we need a harmonized scheme such as the one described in this paper to make sense of the multitude of AI terminology for the types of approaches reported in the health care AI literature. The systems-based perspective in this review provides the means for harmonizing AI life cycles and incorporating quality standards through different maturity stages, which could help advance health care AI research by scaling up to clinical validation and implementation in routine practice. Furthermore, we need to move toward explainable AI approaches where applications are based on clinical models if we are to move toward later stages of AI maturity in health care (eg, clinical validation, and implementation) [74].

### Proposed Guidance

To improve the quality of future health care AI studies, we urge AI practitioners and researchers to draw on published health care AI quality standard literature, such as those identified in this review. The type of quality standards to be considered should cover the trustworthiness, methodological, reporting, and technical aspects. Examples include the NAM and EUC AI frameworks that address trustworthiness and the EQUATOR network with its catalog of methodological and reporting guidelines identified in this review. Also included are the Minimum Information for Medical AI Reporting guidelines and technical ISO standards (eg, robotics) that are not in the EQUATOR. Components that should be standardized are the AI ethics, approaches, life cycle stages, and performance measures used in AI studies to facilitate their meaningful comparison and aggregation. The technical standards should address such key design features as data, interoperability, and robotics. Given the complexities of the different AI approaches involved, rather than focusing on the underlying model or algorithm design, one should compare their actual performance based on life cycle stages (eg, degree of accuracy in model development or assessment vs outcome improvement in implementation). The summary list of the AI quality standards described in this paper is provided in Multimedia Appendix 9 for those wishing to apply them in future studies.

### Implications

Our review has practice, policy, and research implications. For practice, better application of health care AI quality standards could help AI practitioners and researchers become more confident regarding the rigor and transparency of their health care AI studies. Developers adhering to standards may help make AI approaches in domains less of a black box and reduce unintended consequences such as systemic bias or threats to patient safety. AI standards may help health care providers better understand, trust, and apply the study findings in relevant clinical settings. For policy, these standards can provide the necessary guidance to address the broader impacts of health care AI, such as the issues of data governance, privacy, patient safety, and ethics. For research, AI quality standards can help advance the field by improving the rigor, reproducibility, and transparency in the planning, design, conduct, reporting, and appraisal of health care AI studies. Standardization would also allow for the meaningful comparison and aggregation of different health care AI studies to expand the evidence base in terms of their performance impacts, such as cost-effectiveness, and clinical outcomes.

### Limitations

Despite our best effort, this umbrella review has limitations. First, we only searched for peer-reviewed English articles with “health” and “AI” as the keywords in MEDLINE and Google Scholar covering a 36-month period. It is possible to have missed relevant or important reviews that did not meet our inclusion criteria. Second, some of the AI quality standards were only published in the last few years, at approximately the same time when the AI reviews were conducted. As such, it is possible for AI review and study authors to have been unaware of these standards or the need to apply them. Third, the AI standard landscape is still evolving; thus, there are likely standards that we missed in this review (eg, Digital Imaging and Communications in Medicine in pattern recognition with convolutional neural networks [75]). Fourth, the broader socioethical guidelines are still in the early stages of being refined, operationalized, and adopted. They may not yet be in a form that can be easily applied when compared with the more established methodological and reporting standards with explicit checklists and criteria. Fifth, our literature review did not include any literature reviews on LLMs [76], and we know there are reviews of LLMs published in 2023 and beyond. Nevertheless, our categorization of NLP could coincide with NLP and DL in our Euler diagram, and furthermore, LLMs could be used in health care via approved chatbot applications at an early life cycle phase, for example, using decision trees first to prototype the chatbot as clinical decision support [77] before advancing it in the mature phase toward a more robust AI solution in health care with LLMs. Finally, only one author was involved in screening citation titles and abstracts (although 2 were later involved in full-text review of all articles that were screened in), and there is the possibility that we erroneously excluded an article on the basis of title and abstract. Despite these limitations, this umbrella review provided a snapshot of the current state of knowledge and gaps that exist with respect to the use of and need for AI quality standards in health care AI studies.

### Conclusions

Despite the growing number of AI standards to assess the quality of health care AI studies, they are seldom applied in practice. With the recent unveiling of broader ethical guidelines such as those of the NAM and EUC, more transparency and guidance in health care AI use are needed. The key contribution of this review was the harmonization of different AI quality standards that could help practitioners, developers, and users understand the relationships among AI domains, approaches, life cycles, and standards. Specifically, we advocate for common terminology on AI life cycles to enable comparison of AI maturity across stages and settings and ensure that AI research scales up to clinical validation and implementation.

## Appendix

Multimedia Appendix 1 PRISMA (Preferred Reporting Items for Systematic Reviews and Meta-Analyses) checklist.

Multimedia Appendix 2 PubMed search strings.

Multimedia Appendix 3 Characteristics of the included reviews.

Multimedia Appendix 4 List of excluded reviews and reasons.

Multimedia Appendix 5 Quality of the included reviews using Joanna Briggs Institute scores.

Multimedia Appendix 6 Health care artificial intelligence reviews by life cycle stage.

Multimedia Appendix 7 Quality standards found in 10% of unique studies in the selected reviews.

Multimedia Appendix 8 Quality standard–related issues mentioned in the artificial intelligence reviews.

Multimedia Appendix 9 Summary list of artificial intelligence quality standards.

## Abbreviations

AI: artificial intelligence
CONSORT-AI: Consolidated Standards of Reporting Trials–Artificial Intelligence
COREQ: Consolidated Criteria for Reporting Qualitative Research
DL: deep learning
EQUATOR: Enhancing the Quality and Transparency of Health Research
EUC: European Union Commission
FDA: Food and Drug Administration
HIPAA: Health Insurance Portability and Accountability Act
ISO: International Organization for Standardization
JBI: Joanna Briggs Institute
LLM: large language model
ML: machine learning
NAM: National Academy of Medicine
NLP: natural language processing
PRISMA: Preferred Reporting Items for Systematic Reviews and Meta-Analyses
SPIRIT-AI: Standard Protocol Items: Recommendations for Interventional Trials–Artificial Intelligence
