# Supplementary material for: AI Quality Standards in Health Care: Rapid Umbrella Review
Source: J Med Internet Res. 2024 May 22;26:e54705. doi: 10.2196/54705 (PMC11153979; doi:10.2196/54705)
Supplement: Multimedia Appendix 2 [file jmir_v26i1e54705_app2.docx]

Appendix 2 – Pub Med Search Strings

**Database: Ovid MEDLINE(R) <1946 to December Week 2 2022>**

Search Strategy:

--------------------------------------------------------------------------------

1 ("artificial intelligence" or "AI").tw. [January 1st, 2020 to December 31, 2022]

(40745)

2 "health".tw. [January 1st, 2020 to December 31, 2022] (1986832)

3 "systematic review".tw. [January 1st, 2020 to December 31, 2022] (195162)

4 1 and 2 and 3 (143)

***************************

and Ovid_Search2 is:

Database: Ovid MEDLINE(R) <1946 to December 31 2022>

Search Strategy:

--------------------------------------------------------------------------------

1 (("artificial intelligence" or "AI") and "health" and "systematic review").tw.

[January 1st, 2020 to December 31, 2022] (143)

Both Ovid searches returned the same results. We wanted to see if there was any difference between having 1 long search term, or "having" 3 shorter searches.
