# Supplementary material for: AI Quality Standards in Health Care: Rapid Umbrella Review
Source: J Med Internet Res. 2024 May 22;26:e54705. doi: 10.2196/54705 (PMC11153979; doi:10.2196/54705)
Supplement: Multimedia Appendix 6 [file jmir_v26i1e54705_app6.docx]

Appendix 6. Healthcare AI Reviews by van de Sande’s Lifecycle Phases

| **Review** | **Objectives** | **Studies** | **Topics** | **Approaches (source from original review)** | **Domains** | **Original lifecycle stages in reviews** | **Mapping to van de Sande Lifecycle phases** | **Quality Standards*** |
| --- | --- | --- | --- | --- | --- | --- | --- | --- |
| Abd-Alrazaq  2020  [36] | Explore AI use in COVID pandemic | 82 | Public Health, Risk Prediction, COVID-19, | CNN, SVM, RF, DT, LoR, RNN, ANN (unspecified), TL, AE, DNN, KNN, LASSO, Polynomial Neural Network, MLP, ADQN, AB, ARIMA, BA, BERT, CBOW, EM (Eureqa Modeling), GA, GAN, GLM, HAM, LDA, LIR, LM, MTDP (Multi-Task Deep Model), NB, PS, RL, SM, TSF, USEL, VAR (table 3, Appendix 5) | Data Science with NLP | Development  Validation | Mapped to phases 0, I and II | Not mentioned  *Helsinki declaration* |
| Adamidi 2021  [37] | Review AI for COVID screening, diagnosis & prognosis | 101 | Public Health, COVID-19, screening, diagnosis, prognosis | AB, ARMED, BE, BNB, CNN, CRT, CoxPH, ET, GBDT, GBM, GDCNN, GFS, GNB, Inception-RNN, KNN, L1LR, LASSO, LDA, LSTM, MLP, MLR, MRMR, NLP, NB, Nadam, RBF, RFE, RNN, SMOTE, SRLSR  (Section 3B, Table 1, Table 2, Table 3, Table 4, Table 5) | Data Science | Data Preprocessing  Development  Validation | Mapped to phases 0, I and II. | PROBAST  *TRIPOD, FDA-SaMD, STROBE* |
| Barboi 2022  [38] | Compare ML with severity of illness scores for ICU mortality | 20 | Prediction, mortality, ICU | ANN-ELM, DT, ELM, Ensemble LSTM, ESICULA, GA, GBM, KNN, LASSO, LR Ensemble SL, LSTM, Multivariate LR, NN, NN-ELM, RF, RNN, SGB, SVC, SVM, Univariate LR, XGB (Table 3, Table 4) | Data Science with NLP | Data Preparation  Model Training  Validation | Mapped to phases 0, I and II | PROBAST |
| Battineni 2022  [39] | Analyze AI role in development of biobanks | 18 | Biobanks | CNN, SFCN | Data Science, Computer Science | Development,  Performance Assessment | Mapped to phases 0, I maybe II? | NOS |
| Bertini 2022  [40] | Summarize ML that predict pregnancy complications | 31 | perinatal, complications | AB, ANN, DT, EN, GAM, GAuM, GBM, HRF, J48, LDA, LiR, LoR, MRF, MLP, MLR, NB, NN, RBN, RF, SGAM, SVM, XGB (Table 3) | Computer Science | Development  Validation | Mapped to phase 0, I and II with need for phase III clinical testing | CASP |
| Bhatt 2022  [41] | Examine AI in mHealth for remote patient monitoring & disease management | 37 | mHealth, disease management | DL,FL | Data Science | Development  Assessment? | Mapped to phases 0, I | Not mentioned |
| Buchanan 2020  [42] | AI trends & implications on nursing admin, practice & research | 118+13 | administration, clinical practice, policy, research | ML, SAR, CDSS, chatbots | Data Science, Robotics | Conceptual Research  Development  Testing  Evaluation | Implied need for nurses to be involved with all phases from need to design to implementation | Not mentioned  *ISO1-3482, EU-SPARC, ISO/TC299, COREQ* |
| Buchanan 2021  [43] | Summarize influences of AI on nursing education | 27 | education | ML, virtual avatar applications, chatbots, wearable armband w/ ML, predictive analysis, virtual and augmented reality, smart homes, robots | Data Science, Robotics | Design  Implementation  Evaluation | Implied need for nurses to be part of co-design at all stages. Strong educational foundation in AI principles is first step | Not mentioned |
| Chew 2022  [44] | Provide overview of perceptions & needs of AI to increase adoption in healthcare | 26 | general | Chatbots, Weak AI, image recognition, AI diagnosis, NLP, predictive modeling | Data Science with NLP, Robotics | Knowledge Persuasion  Decision  Implementation  Confirmation | Needs based analysis is recommended before development of AI systems | Not mentioned |
| Choudhury 2020a  [45] | AI/ML in geriatric chronic diseases | 35 | patient safety outcomes | ANN, BICMM, BNC, C4.5, CPH, DBN, DCNN, DT, EMLA, GNB, IFAST, KL, KNN, LASSO, LDA, LP, L1-LR, LR, LSR, M2TL, MDR, MLFFB, mSVM, MKSVM, NB, PCA, PSO-SVM, RF, RP, SOM, SVM-RBF, SVM, XGB (Table S1) | Data Science | Model Development  Training  Validation | Mapped to phases 0 and I | ISO/IEC CS 23053, ISO/WD TR 22100-5, NIST, Laskai, OECD-AI (p464,467) ** |
| Choudhury 2020b  [46] |  | 53 | geriatrics, disease management | AUC, AI, BCP-NN, BCPNN, BNM, BCT, BiLSTM, C4.5, CARD, CART, CC, CHAID, CNN, CRF, CRFNN, CSS, DEWS, DT, ET, J48, KNN, LASSO, LR, LRM, LSTMRNN, MEWS, MLP, MMD, MT, NB, NN, NN-BP, NLP, RDAC, RF, RNN, SAC, SELF, SVR, SVM, XGB | Data Science with NLP | Development  Validation | Mapped to phases 0 and I | TRIPOD, TRIPOD-ML (cited but not used in AI studies; cited NIST standards p23)  *FAERS, MedEx, RxNorm, MedDRA, PCONet, MADE* |
| Eldaly 2022  [47] | Use of AI in lymphedema prevention, diagnosis & management | 15 | prevention, diagnosis, disease management | ANN, ANFIS, Chatbots, DT, EML, FLDA, FM, GBRM, GLM, GPRM, KNN, MARS, MEENM, NB, NLP, RF, Robotics, SL, SRE, SVM (Discussion) | Data Science, Robotics | Development implied  Validation needed | Mapped to phase 0 and I | Not mentioned |
| Glaz 2021  [48] | Summarize ML-NLP for mental health methodological & technical terms | 58 | mental health | C4.5, CRA, CUI, DT, KM, LDA, LiR, LoR, LSA, NB, NN, RF, SentiAna, SVM, TF-IDF | Data Science with NLP, Computer Science | Preprocessing  Classification | Mapped to phases 0 and I | Not mentioned  *UMLS* |
| Guo 2021  [49] | Examine if AI applications integrated heterogeneous data for COVID modeling | 794 | Prediction, diagnosis, prognosis, COVID-19 | 3DQI, ACNN, AB, AI, ANN, ANFIS, ARIMA, BFDA, BGFS-PNN, BN, cGAN, CLHC, CNN, CPC-NN, CPH, DE, DML, DNN, DSA-MIL, DT, DoL, EDEM, EN, ET, FDA, GB, GLM, GNB, HC, K%BD, KM, k-NN, KG, LASSO, LDA, LB, LiR, LR, LSTM, MA-CLR, MLP, NB, NC, NLP, OneR, PCA, PCR, PLS, PLSR, PSO, QDA, RBF, RF, RT, SC, SGD, SHAP, SOM, SVM, U-Net, XGB, ZeroR | Data Science | Not Mentioned | Mapped to phases 0 and I | Not mentioned |
| Hassan 2021  [50] | Identify optimal predictors of infection & sepsis | 17 | Prediction, sepsis | InSight, LASSO, LoR, MCRM, MLR, NNA, RF, RNN, SRM, SVM, TABN, ULR (Table 3) | Data Science and Computer Science | Development  Training  Validation | Mapped to phases 0, I and II | TRIPOD, NOS |
| Huang 2022  [51] | Develop AI telemedicine to monitor self-isolation & COVID progression | 13 | telemedicine, monitoring, COVID-19 | CNN-TF, IRRCNN, IoT-based Wearable Monitoring Device, NVHODL, SVM, Wearable Biosensors (Table 1) | Data Science | Development  Training  Validation | Mapped to phases 0 and I | Not mentioned |
| Kaelin 2021  [52] | Develop AI telemedicine to monitor self-isolation & COVID progression | 94 | Pediatric, rehabilitation | NLP, ML, computer vision, robotics (table 1 p6) | Data Science, Computer Science, and Robotics | Development  Testing | Mapped to phases 0 and I | Not mentioned  *HIPAA, FERPA, COREQ* |
| Kirk 2021  [53] | Provide overview of ML in precision nutrition | 60 | nutrition | BC, CNN, DL, DT, EM, GBR, HC, IoT, KBHDNN, KM, KNN, LiR, LoR, ML, NLP, DRNN, RF, Regression, SVM, WS | Data Science and Computer Science | Development  Training, Testing  Validation, Evaluation | Mapped to phases 0, I, II? | Not mentioned (not even PRISMA) |
| Loveys 2022  [54] | Review effectiveness of AI interventions in old people receiving LTC services | 31 | geriatrics, interventions | AI-enhanced robots, social robots, environmental sensors, wearable sensors (Table 1) | Data Science, Computer Science, and Robotics | Development | Mapped to phases 0, I | Revised Cochrane Risk of Bias for RCT, cluster RCT & Non-RCT (ROBINS-I) – figure 2 |
| Morch 2021  [55] | Review use & ethics of of AI in dentistry | 178 | dentistry, ethics | DL, DSP, ML, NN | Data Science | Development  Validation |  | Mentioned need for SPSIRT, TRIPOD, used 2018 Montreal Declaration as AI ethical framework (p1458) |
| Payedimarri 2021  [56] | Evaluate AI & ML public health interventions to contain COVID | 8 | public health, interventions, COVID-19 | ABS, LiR, NN, TOPSIS | Data Science | Development  Validation | Mapped to phases 0, I | Not mentioned |
| Popescu 2022  [57] | Review of AI to detect melanoma | 134 | Cancer, prediction melanoma | ABC, Autoencoder, CNN, Combined Networks, DCNN, DCGAN, DDGAN, Encoder/Decoder, FBDA, FCNN, GAN, HLPSO, KM, KNN, LAPGAN, LiR, MNN, NB, NN, Perceptron, RCNN, RF, RVM, SPGGAN, SVM, SNN, TAB, TrCSVM (Table 3 & 4) | Data Science, Computer Science | Preprocessing  Segmentation  Classification | Mapped to phases 0, I, II | Not mentioned |
| Rahimi 2021  [58] | Review AI in community based primary health care | 90 | primary care, diagnosis, disease management | AL, AR, BN, COBWEB, CH, DM, DT, ES, FL, GBT, Hybrid, KNN, KR, LASSO, LGMM, LoR, MM, MCA, NLP, NN, RF, RIPPER, SVM (Fig 3, pg. 8) | Data Science with  NLP | Development  Testing, Validation  Implementation | Mapped to phases 0, I, II | PROBAST |
| Sahu 2022  [59] | Review prediction models for early neonatal sepsis | 10 | Detection, neonatal, sepsis | AR-HMM, LiR, LoR, MLoR, NN, FWSLRA (Table 1-3) | Data Science | Development  Training, Testing  Validation | Mapped to phases 0, I, II | CHARMS, PROBAST |
| Sapci 2020  [60] | Evaluate AI training & use to enhance learning experience | 26 | education | AI, DL, ITS, ML, NLP, VR | Data Science with NLP, Computer Science | Development, Validation, Training, Evaluation  Implementation | Mapped to phases 0, I, II, III | Not mentioned  *AMA-Augmented Intelligence* |
| Seibert 2021  [61] | Synthesize AI literature in nursing care | 292 | ethics | APS, ML, ES, Hybrid, NLP (Table 3 & 4) | Data Science, Computer Science, and Robotics | Development, Training Testing, Validation  Implementation  Evaluation | Mapped to phases 0, I, II, III | Risk of bias; levels of evidence from I to VII & not applicable  *SWE, OGC, SOS, COREQ, STROBE* |
| Syeda 2021  [62] | Review AI to fight COVID | 130 | epidemiology, diagnosis, disease progression, COVID-19 | ANN, BiGAN, CNN, DT, DL, ENN, KNN, LoR, LSTM, MLP, ML, PNN, RNN, RF, SEIR, Simulation, SORD, STN, SVM, XGBoost (appendices 2-4) | Data Science | Development, Training, Validation  Evaluation | Mapped to phases 0, I, II | Not mentioned |
| Talpur 2022  [63] | Review ML and dental caries association | 12 | Dentistry, caries | ADA-NN, ANN, CNN, F-CNN, FFBP-ANN, HLP-BP, KNN, LM-NN, LoR, R-CNN, RF, RNN, SVM (Table 2, Figure 6) | Data Science | Development, Training, Testing, Validation | Mapped to phases 0, I, II | Risk of bias assessment p3, no other standards mentioned |
| Velez-Guerrero 2021  [64] | Review AI mobile robotic exoskeletons in upper limb rehab | 30 | rehabilitation | AL, ANN, AFM, ATC, AFC, PSO, FL, AFPIDC, ASM, KNN, RL, PPO, HSVM, BPNN, FSMC, MMVR (Table 2) | Data Science, Computer Science, and Robotics | Development  Training, Testing  Validation | Mapped to phases 0, I, II | Need standardized protocol for clinical evaluation, FDA regulatory standards |
| Welch 2022  [65] | Examine wearables in psychiatric assessment of child-adolescent patients | 19 | Diagnosis, pediatrics, psychiatry | ML, wearable biosensors | Data Science with NLP, Computer Science | Trial  Evaluation | Mapped to phases 0, I, II | Not mentioned |
| Zhao 2021  [66] | Identify ethical issues in infectious disease outbreak surveillance | 29 | public health, surveillance, COVID-19 | AI, AR, ML, physiologic monitoring, sensory technologies, speech recognition technology, VR, wearable devices | Data Science | Development  Implementation | Mapped to 0, I, II, III, IV? | MMAT for study quality  Asadi framework for ethics  *SORMAS, STARD* |
| Zheng 2022  [67] | Synthesize NLP literature to identify hypoglycemia in EHR notes | 8 | Detection, hypoglycemia | ML or rule-based NLP | Data Science with NLP (rule-based) | Development  Validation | Mapped to 0, I, II | Not mentioned |
| Zidaru 2021  [68] | Explore patient-public involvement for AI in mental health | 144 | mental health | ML, NLP, sentiment analysis, VR, wearable biosensors | Data Science | Public engagement  Planning, Development, Implementation, Evaluation, Diffusion | Mapped to 0, I, II, III, IV? | Need standards for evaluating safety, outcomes, acceptability, explainability, inclusive design  *EU AI-Watch, FDA-SaMD, EUQATOR, WEF-WHO governance, CCC AI roadmap*  *ISO/IEC/IEEE-12207* |

Legends: phase 0-preparation prior to model development, I-AI model development, II-assessment of AI performance and reliability, III-clinical testing AI, IV-implementing and governing AI. * *italicized entries* are AI quality standards mentioned only in the original studies in the reviews
