# Supplementary material for: AI Quality Standards in Health Care: Rapid Umbrella Review
Source: J Med Internet Res. 2024 May 22;26:e54705. doi: 10.2196/54705 (PMC11153979; doi:10.2196/54705)
Supplement: Multimedia Appendix 8 [file jmir_v26i1e54705_app8.docx]

Appendix 8. AI Quality Standard Related Issues Mentioned in Reviews

| **Review** | **Objective** | **Themes** | **Quality Related Issues** |
| --- | --- | --- | --- |
| Abd-Alrazaq  2020  [36] | Explore AI use in COVID pandemic | Reporting standards  Federated learning  Explainability  Education, Ethics | Many proposed research not yet clinically accepted; most rewarding research will be on promising methods beyond COVID; mobile phones have potential but need energy-efficient & federated learning; NLP to communicate non-pharmaceutical interventions unexplored with public data; need standardized reporting protocols, ethics & explainable AI paired with public education (p12) |
| Adamidi 2021  [37] | Review AI for COVID screening, diagnosis & prognosis | Explainability  Federated learning  Trust  Transparency Bias | Application in clinical may be problematic due to use of single data source. Most prediction models trained on specific localized dataset; evaluation of AI techniques & importance of predictors cannot be discerned through meta-analysis. Evaluation of explainability difficult due to lack of uniform adoption of interpretability assessment criteria; also need federated learning (p2847). Integration of explainability modalities in developed models can enhance human understanding on reasoning process, maximize transparency & embellish trust toward model use in clinical practice (p2848). |
| Barboi 2022  [38] | Compare ML with severity of illness scores for ICU mortality | Reporting standard  Performance measures  Transparency | Reporting methodology is incomplete, non-adherent to current recommendations. Lack of consistent reporting of measures of reliability calibration, discrimination & classification or probabilistic estimates make comparison of models challenging – could not assess superiority or inferiority of ML-based ICU mortality prediction compared with traditional models. For model developers (1) state if models are for clinical practice, (2) provide transparency of clinical setting for data source & model development steps, validate model externally to ensure generalizability, (3) report model performance measures including measures of discrimination, calibration & classification, and attach explainer models to facilitate interpretability (p17) |
| Battineni 2022  [39] | Analyze AI role in development of biobanks | Personalized care | Many ML methods have been developed and used to improve analytics challenges across studies of complex human diseases. Biobanks and AI could be supportive for clinical practice and choosing best AL algorithms for developing prognosis models (p8). Transformation of personalized care by coupling biological data with EHR, Adding AI algorithms into personalized patient data can help answer questions on genetic variation impact on human health (p9) |
| Bertini 2022  [40] | Summarize ML that predict pregnancy complications | Ethics  Performance measures  Reporting standards  Clinical utility | Ethical dilemmas for biases. Different output variables not comparable. Reports of predictive accuracy often incomplete with multiple methods. Difficult to determine best prediction methods since they do not have same inputs, type of records and samples. ML algorithms are black boxes, need to understand internal functioning of model, granting interpretability and transparency (p12); clinicians would need to know which variables are involved in diagnosis prediction (p13). Studies have different baselines, variables and separate complications difficult to compare. Models should be used in hospital or health services for pregnant women, and with prospective studies & additional population studies to assess clinical utility of model for real world (p13) |
| Bhatt 2022  [41] | Examine AI mHealth for remote patient monitoring, disease management | Trust  Explainability  Federated learning  Transparency  Rules on wearables | Confluence of AI & mobile wearable technology led to increased mhealth use, & mhealth & telehealth provide noncritical care (p7). Advances in FL and XAL could (a) expand mHealth services, (b) build consensus on rules governing wearable tech use, (c) organizations should promote use of mHealth in digital health & public safety campaigns – need to protect patient privacy, increase trust in smart wearables (p6). XAI needed to increase acceptability & understanding of ML; need for transparency reason for non-adoption |
| Buchanan 2020  [42] | AI trends & implications on nursing admin, practice & research | Co-design | Nurses may spend less time with patients or be given larger workload. Need to monitor to ensure AIHT can augment & enhance care not replace it –nursing leadership needed to preserve person-centered compassionate care (p6). New opportunities to work as care coordinators. Policy – new policies to support integration of AIHT into practice to promote safety & quality care (p7). Admin - assist automated analysis of patient data, scheduling, reducing documentation burden, triaging patients, streamlining workflow, improving efficiency & accuracy of patient care, give rise to new nursing care delivery models and new admin roles (p7). Research – how AI influence compassionate care, identify best practices for optimal implementation, need for co-design of AI, establish nursing informatics officers to guide procurement, design & implementation of AI. |
| Buchanan 2021  [43] | Summarize influences of AI on nursing education | Co-design  Privacy  Equity, Ethical  Social justice | Education should be provided in academic and clinical practice settings through professional development opportunities. Advanced technology will change traditional nursing processes and ways of knowing. Nurse educators need to reflect on past practices and transition toward new ways of engaging students. Need to balance human caring needs with technological AI advancements. Identify educational requirements and core competencies needed to integrate AIHT into nursing practice & as a tool in nursing education (p7) |
| Chew 2022  [44] | Provide overview of perceptions & needs of AI to increase adoption | Transparency  Trust, Safety,  Technology maturity  Education | Table 2 perceptions – available on demand & user-friendly, efficiency, price, lack of trust in data privacy, patient safety, technology and concerns over full automation (p5-9); Table 3 needs & mitigation strategies – need for transparency, credibility & regulation; lack of personalization & customizability, perceived empathy & personification; design, user experience & interconnectedness with other devices, educating public on AI capabilities (p10-13); mitigation strategies: data privacy- transparency in how data will be used; safety –regulation, credibility, transparency, liability; technology –integration, standardization, sustainability; technology maturity. Improve adoption: enhance personalization & customizability, enhancing empathy & personification of AI-enabled chatbots and avatars, enhancing user experience design, interconnectedness with other devices, education public on AI capabilities (p1). Challenges lies in extent to which AI is tailored according to individual unique preferences. Prior needs-based analysis before development of AI systems (p15). Enhance transparency over predictive accuracy and info sources (p15) |
| Choudhry 2020a  [45] | Understand current use of AI/ML in geriatric clinical care for chronic diseases | Standardization  Safety  Governance  Context-specific | Lack of standardization in ML evaluation, framework for data governance. Development of standards underway (a) ISO/IEC CS 23053 Framework for AI systems using ML; (b) ISO/WD TR 22100-5 Safety of machinery relationship with ISO 12100-part5: implications of embedded AI-ML; (c) Royal Society of Great Britain acknowledged goal of data governance as legal & ethical norms of conduct & practices that govern collection, storage, use and transfer of data; (d) US ONC released draft 2020-25 Federal HIT strategic plan to develop health IT infrastructure to include interoperability standards; (e) Executive Order 13859 directing Federal agencies to develop a plan to ensure AI/ML standards; (f) China MST published framework & action guidelines – Principles for a New Generation of AI: Develop responsible AI ; (g) Office of President of Russian Federation released a national AI strategy & Russian AI policy on OECD AI Policy Observatory; - ML algorithms in healthcare are context-specific – healthcare requires standards (governance) that are tailored toward its goal (p466)  ML estimates prognosis are based on static data and algorithms. Need to incorporate models into clinical workflow to match the measure and underlying disease trajectory to patient’s individual situation. (p466). Studies implementing ML must consider baseline risk for their outcomes for patients with higher or lower risks than general population. ML models should report short-, mid- and long-term effects of models’ recommendations on patient health outcomes. ML models or tools are usually developed and tested in specific settings, which potentially limit its measure’s validity in other contexts –might generate performance measures lower than existing validated tools. Lack of AI standards, data governance, and integrated global healthcare database affect ability to integrate ML into clinical workflow (p467) |
| Choudhry 2020b  [46] | Identify & analyze quantitative studies on AI in clinical patient safety outcomes | Standardization  Evaluation measures  Reporting Standards  Safety, Privacy  Regulation, Benchmark data  Education | Issues: (a) Lack of standardized benchmark among reported AI models –safety outcomes do not necessarily correlate to AI performance measures. Need to be scrutinized against standard of care by clinicians or clinical gold standard. Need to standardize AI in healthcare, need to be tailored for specific purpose otherwise may hamper patient safety; (b) United States President issued an Executive Order EO 13859 to develop AI standards. NIST –standardized AI benchmark can serve as a mechanism to evaluate & compare AI systems. FDA acknowledged AI standardization can assure ongoing algorithm changes follow pre-specified performance objectives & use a validation process that ensure safety; (c) reporting of AI model studies not standard, making comparison of algorithms across studies challenging; need to compare on same data representative of target population also same evaluation measures – standardized reporting of AI studies needed, e.g. TRIPOD-ML is in development for ML prediction algorithms; (d) also need to determine important AI evaluation measure, which ones should be measure in given context (p22); (e) drug safety followed by analysis of clinical report most common area of interest for AI to address clinical level patient safety concerns; identified by HIPAA –regulations identify risk analysis as part of safeguard requirements to improve patient safety; (f) gold standard needed for various contexts/disease/problems against AI performance can be measured – common independent test using unenriched representative sample not available to training algorithms; (g) standard framework needed to guide clinicians in interpreting clinical meaning of AI evaluation measures before integrating into clinical workflow; with quantifiable measure of AI demonstrating analytic performance and impact on patient safety – short & long term, reliability, domain-specific risks, and uncertainty; (h) regulatory bodies should invest in data infrastructure such as standardization of EHRs and integration of different health database; NIST -> standardized data can make training data more visible and usable to users; need to train AI on critical adjustments made by clinicians to adapt to different conditions (p23) |
| Eldaly 2022  [47] | Explore AI in lymphedema prevention, diagnosis & management | Technology feasible but need validation | AI based system was able to predict & diagnose lymphedema accurately. Lymphatic rehab exercises can be challenging to patients, motion capture systems not practical for clinical use as they require patient to wear suit and perform exercise in room with cameras and are expensive. Robotic assisted surgery is safe & effective for staging & treatment of cervical & endometrial cancer, no different or safer for postoperative complications, lacks RCT for strong evidence (p240). |
| Glaz 2021  [48] | Summarize ML-NLP for mental health | Biased data sources  Ethics  Privacy  Transparency | New paradigm to reconsider standard methodology – formulate sound hypothesis, define objective, collect results to uphold or reject hypothesis. Limitations are bias data sources, cultural/ethnic differences. Few meaningful contributions to clinical practices. There may be new ways for people to express mental illness. Raises ethical issues & require prudence. Intro of computer change physician-patient relationship & promotes a new clinical model involving third party. Precautions to avoid clinical abuse p13. Ethical concerns in balancing beneficence & respecting confidentiality. Protect privacy to inform patients – (1) algorithm can be wrong, (2) algorithm data highly sensitive, (3) algorithm data might recommend actions not immediately apparent, (4) algorithm might prompt unnecessary intervention from provider. Need to explain patient the main characteristics & why they require recommendations (p14) |
| Guo 2021  [49] | Examine if AI applications integrated heterogeneous data for COVID modeling | Data integration  Privacy  Multilevel AI framework | 7 key AI areas: disease forecasting, imaging-based diagnosis & prognosis, early detection & prognosis, drug repurposing & early drug discovery, social media, genomic transcriptomic & proteomic data (p2050). Wide range of ML and DL algorithm used for modeling, with close to half in imaging analysis for diagnosis. Lack of data integration from heterogeneous sources. Leading to confounding bias. Some data not available or due to privacy reasons. Could have included public data on risk factors. Need for multilevel AI framework that supports analysis of heterogeneous data from different sources (p2063) |
| Hassan 2021  [50] | Identify optimal predictors of infection & sepsis | Model & predictor classification  Model performance | ML can help sepsis prediction but not recommendation to reduce risk of sepsis; most models used old definitions of sepsis that limited specificity & sensitivity. Type of sepsis predictors & how sepsis is defined can improve predictive power & timeframe of ML learning algorithm. Classifying predictors as modifiable & non-modifiable can support development of algorithms that provide recommendations to reduce risk of sepsis (p8) |
| Huang 2022  [51] | Develop AI telemedicine to monitor self-isolation of COVID progression | Trust  Transparency  Engagement  Performance | Telemedicine challenges –privacy, security, data format & management, synchronization, over-tracking, development & implementation guidelines, user engagement (p7). Trust, transparency, engagement, device accuracy (p8) |
| Kaelin 2021  [52] | Examine how AI is integrated in pediatric rehab interventions | Context  Personalized care | 3 major gaps –lack of remote participation-focused AI interventions, individual goal setting integrated in interventions, interventions tailored to individually reported participation needs of children, youth or families; should consistently report on socioeconomic background, parental education, race & ethnicity for diversity of study sample (p8) |
| Kirk 2021  [53] | Provide overview of AI in precision nutrition | Integration  Preprocessing  Online learning  Performance  Cost-effective  Explainable | Current review did not pay attention to ML for preprocessing of data e.g., feature selection or dimensionality reduction as separate process to main outcome. Some studies did not test efficacy of their findings in impacting human health. Some data collection methods rely on questionable efficacy such as self-report. Some PN models do not consider socio-demographics such as gender, age, drugs, sleep quality. Performance should be tested with rigorous large-scale trials & for cost-benefits. None made use of online learning methods that continually update itself in response to continuous inflow of new data to remain current. PN must prove itself to be effective & worth investment. PN must be embrace explainable AI. Need for professionals to understand how models have arrived a conclusion and convey to patients (p23-24) |
| Loveys 2022  [54] | Review effectiveness of AI interventions in old people receiving LTC services | Ethics  Privacy  Data protection | Ethical considerations – older people attached to robots & experienced distress when separated. Surveillance concerns with sensors; communication to LTC consumers needed for data privacy & protection process; protection against unanticipated harm, timing of installation should take place before dementia onset and progression. Clinical trials needed, screen-based AI interventions needed over robots in resource limited settings. More studies needed on assistance in DAL & managing chronic conditions(pe295) |
| Morch 2021  [55] | Review use & ethics of of AI in dentistry | Ethics | Most studies validated internally; need ethics guidelines; recommended description of broader impact of work including ethical & societal consequences, positive & negative outcomes, making system more explainable; used 2018 Montreal Declaration as an AI ethics framework (p1458); table with 10 ethical issues –prudence, equity, privacy & intimacy, responsibility, democratic participation, solidarity, diversity inclusion, well-being, respect for autonomy, sustainable development (p1456) |
| Payedimarri 2021  [56] | Evaluate AI & ML public health policy interventions to contain COVID | Context & time dependency | Strategies included quarantine, lockdown variations & social distancing. Comparison of different families of modeling methods difficult, dependent on kind of data, availability of analytic tools, & context. ML learns abstract behavior of system from observations to make predictions; no one model is better or worse than another p8-9. |
| Popescu 2022  [57] | Review of AI to detect melanoma | Decision fusion | NN as part of AI are increasingly being researched in imaging applications as a support system for diagnosing SL and detecting ME. Use of NN in detection of melanoma may be involved in a support system for dermatologist who decide either a biopsy or another type of lesion. AI & ML could have been useful to help policy makers to better define best strategies for containing COVID since end of first wave. p34-35 |
| Rahimi 2021  [58] | Review AI in CBPHC (community based primary health care) | Reporting standards  User & provider  involvement  SDOH bias  Ethical & legal  Economic benefits  Trustworthiness  Barriers, facilitators | Variabilities in reporting participants, AI methods, analysis, outcomes, & gaps in AI development & implementation p10 – high risk of bias in diagnosis-prognosis studies, performance may not be as optimal as reported. AI not used in admin & operational tasks. Needs to use knowledge, attitudes & behavior theories to expand AI use in clinical decision making; frameworks needed to guide development & implementation of AI in CBPHC. Sex, gender, age and ethnicity rarely considered. Lack of user involvement (p11). Ethical & legal aspects rarely addressed except for privacy & data security; need to clarify need for informed consent; responsibilities for use of AI systems, responsibilities for researcher for development & implementation of AI health literacy programs. Need to demonstrate cost-effectiveness of AI –economic benefits in terms of treatment, time & resource management, mitigation of human error. Barriers & facilitators for implementing AI in practice mostly related to data – data governance, open data directives, and other data initiatives to establish trustworthy mechanisms and services for sharing, reusing & pooling data; data security and privacy laws can limit use of AI & sharing of info (p12) |
| Sahu 2022  [59] | Review prediction models for early neonatal sepsis | External validation | External validation needed to ensure model accuracy. |
| Sapci 2020  [60] | Evaluate AI training & use to enhance learning experience | AI education  AI competencies  Learning experience | Figure 4 – classification category 1 – AI tools to enhance medical education, II – teaching AI as a new competency p9; Figure 5 proposed framework for specialized AI training: (a) medical students to apply AI to improve patient care, discuss implications, compare & evaluate AI tools, (b) informatics students to apply ML algorithm to analyze data, integrate analytics & visualize datasets, (b) computer science students to develop AI programs to solve problems, apply big data analytics; in areas of diagnostics, therapeutics, predictive medicine, learning health systems p11 |
| Seibert 2021  [61] | Synthesize AI literature in nursing care | Privacy, Safety, Benefits, Education, Acceptance,  Ethical, Legal & Social implications | More knowledge needed on benefits & advantages of AI compared with alternative solutions or usual care. Need perspectives & experiences of nurses, care dependents & informal caregivers on AI in nursing care. Need AI effectiveness in real-world scenarios that are nursing care specific in objectives, outcomes & benefits (p13). Need empirical evidence of effectiveness & longitudinal evaluation of AI systems. Education & AI knowledge needed (p14)  Need reporting & requirements on data privacy, safety, technology acceptance, ethical, legal and social implications p1 |
| Syeda 2021  [62] | Review literature on AI to fight COVID | Clinical validation  Explainability  Performance | AI performance might be biased due to lack of adequate sample size, detailed analysis of variable needed to identify COVID progression, sentiment analysis needed with social media content & negative impacts on mental health condition, lacking detail on how AI model predictions were interpreted, comparison of AI model performance not possible due to variation in sample size & data source variations (p9-10) |
| Talpur 2022  [63] | Review ML and dental caries association | Study Quality | Well designed studies are needed to demonstrate the diagnosis of further types of dental caries that are based on progression (chronic, acute and arrested), which shows the severity of caries, virginity of lesion, and extent of caries. (p12) |
| Velez-Guerrero 2021  [64] | Review AI wearable robotic exoskeletons for upper limb rehab | Self-adaptability  Integration, Bias  Explainability  Clinical validation | Need more flexible, adaptable, wearable & lightweight structures, need more info on power consumption of rehab systems & interconnection with outside worlds, collection of patient physiological parameters to improve robot control (p22). Clinical validation needed to support rehab process, need to develop exoskeletons that automatically adapt to environment or user, actively learning from input experiences. Still has poor explainability, explicit or implicit bias, unpredictable when it comes to interaction with human/patient user; need for integration of greater expertise across multiple disciplines (p23-24) |
| Welch 2022  [65] | Examine wearables in psychiatric assessment of child-adolescent patients | Data quality  Benefits | Feature extraction & engineering are key to ML using wearable data, and is dependent on nature of clinical questions & types of measurements from wearables. Lack of RCTs, wide definitions of digital biomarkers used, small participant numbers in studies. Integration of wearable data can facilitate remote monitoring & remote psychiatric services to reduce disparities in mental health care access; need extraction of AI benefits from naturalistic studies e.g. children behavior in daily life at home; provide opportunities to innovate with AI approaches such as reinforcement learning. P9 |
| Zhao 2021  [66] | Identify ethical issues in infectious disease outbreak surveillance | Ethics | Asadi ethical framework – (a) individual level – data ownership, data control, awareness, trust, privacy, self-determination, fear; (b) organizational level – data quality, data sourcing, data sharing disclosure, algorithmic decision making, presentation, ethical capability, ethical culture, ethical governance; (c) societal level – power, social awareness, surveillance, principles & guidelines, authority, climate (textbox 1 p12-13) |
| Zheng 2022  [67] | Synthesize NLP literature to identify hypoglycemia in EHR notes | Ability to aggregate results?  Reporting standards | Heterogeneity of reported results prevented estimated of pooled incidence and prevalence of hypoglycemia in diabetes using NLP algorithms. ML & DL based algorithms have been developed but not applied in clinical research Ip12) |
| Zidaru 2021  [68] | Explore patient-public involvement for AI in mental health | Public engagement  Ethics & Safety  Trust, effective, fair  Design justice | See figure 3 on p1083 – 4 principles of design justice in AI. (a) meaningful & authentic public engagement in all areas of AI to be supported and guided by core principles that AI should sustain, heal, connect & empower people & communities (p1082); (b) Ethical concerns e.g. inequalities, cultural & population biases, safety, acceptability & broader socio-political issues need to be better understood & moderated to focus design on community concern (p1083); (c) AI need to emerge from an accountable, accessible & collaborative process that describes how patients & public have been involved (p1092); (d) AI developers can be informed by and contribute to shared knowledge in design & tools to support design justice (p1084)  Or need to understand effective modes of public engagement in AI context, to examine ethical & safety issues, develop new methods of PPI at every stage from concept design to final review of AI in practice. Principles of design justice can guide this agenda (p1072)  Also see table 2 for subthemes (a) mental health application; (b) ethics – inequalities & population biases, socio-political context, safety & acceptability; (c) public engagements –new contexts, public awareness, preference4 & choices, patient & public trust (p1078-1079) |
